# Bacterial membrane vesicles and phages in blood after consumption of *lacticaseibacillus rhamnosus* JB-1

**DOI:** 10.1080/19490976.2021.1993583

**Published:** 2021-11-07

**Authors:** Kevin Champagne-Jorgensen, Tamina A. Jose, Andrew M. Stanisz, M. Firoz Mian, Alexander P. Hynes, John Bienenstock

**Affiliations:** a Neuroscience Graduate Program, McMaster University, Hamilton, Canada; b Brain-Body Institute, St. Joseph’s Healthcare Hamilton, Hamilton, Canada; c Department of Biochemistry and Biomedical Sciences, McMaster University, Hamilton, Canada; d Farncombe Family Digestive Health Research Institute, Department of Medicine, McMaster University, Hamilton, Canada; e Department of Medicine, McMaster University, Hamilton, Canada; f Department of Pathology and Molecular Medicine, McMaster University, Hamilton, Canada

**Keywords:** Bacteriophage, commensal, extracellular vesicles, interleukin 10, lactobacillus, microbiome, microbiota-gut-brain axis, microvesicle, probiotic, TLR2

## Abstract

Gut microbiota have myriad roles in host physiology, development, and immunity. Though confined to the intestinal lumen by the epithelia, microbes influence distal systems via poorly characterized mechanisms. Recent work has considered the role of extracellular vesicles in interspecies communication, but whether they are involved in systemic microbe-host interaction is unclear. Here, we show that distinctive nanoparticles can be isolated from mouse blood within 2.5 h of consuming *Lacticaseibacillus rhamnosus* JB-1. In contrast to blood nanoparticles from saline-fed mice, they reproduced lipoteichoic acid-mediated immune functions of the original bacteria, including activation of TLR2 and increased IL-10 expression by dendritic cells. Like the fed bacteria, they also reduced IL-8 induced by TNF in an intestinal epithelial cell line. Though enriched for host neuronal proteins, these isolated nanoparticles also contained proteins and viral (phage) DNA of fed bacterial origin. Our data strongly suggest that oral consumption of live bacteria rapidly leads to circulation of their membrane vesicles and phages and demonstrate a nanoparticulate pathway whereby beneficial bacteria and probiotics may systemically affect their hosts.

## Introduction

Intestinal microbiota have extensive and diverse roles in mammalian physiology. Consumption of beneficial microbes, probiotics, and complex communities thereof has been shown to modulate metabolism, immunity, and nervous system function, both locally in the gut and throughout the wider organism.^1,2^ Though several candidate mediators of bacterial activity have been proposed, including conserved molecular features, short chain fatty acids, and bacteria-produced hormones, among others,^2,3^ the mechanisms by which bacteria exert systemic influence are poorly understood.

The bacterium *Lacticaseibacillus rhamnosus* JB-1 has diverse immune- and neuro-activity after oral consumption by mice. It promotes a regulatory phenotype in dendritic cells (DCs) and increases the number of functional regulatory T cells,^4,5^ and is protective in murine models of asthma including systemic inhibition of mast cell activation.^6^ It also inhibits hypothalamic-pituitary-adrenal axis stress responses and reduces anxiety-like behavior while altering GABA receptor expression in the brain.^7^ Many of these behavioral effects appear to be mediated by regulatory T cells^8^ or the vagus nerve,^7^ with regional brain activity occurring via both vagus-dependent and -independent mechanisms within 2.5 h of feeding.^9^ Thus, JB-1, like many other nonpathogenic bacteria, exerts systemic influence despite being constrained to the gut lumen.

Extracellular vesicles (EV) may play a role in bacteria-host communication. EV are membrane-bound nanoparticles that are released by all domains of life.^10^ EV from prokaryotic organisms are produced from the plasma membrane and are often termed microvesicles or membrane vesicles (MV), while eukaryotic EV can be produced by budding off the plasma membrane or by release of intracellular endosomes to form exosomes.^11^ Regardless of source, EV can contain proteins, nucleic acids, and other cargo, and are considered important mediators of intercellular communication.^12^

Bacterial MV appear to play an important role in intestinal bacteria-host interactions. MV from pathogens can deliver immunostimulatory and toxin cargo to host cells and directly support bacterial pathogenesis.^13–16^ By contrast, MV from commensal and beneficial bacteria can influence host metabolism,^17^ protect against intestinal inflammation,^18–20^ and modulate intestinal barrier strength,^21^ among others.^22^

Many gut effects of JB-1 are recapitulated by its MV, including activation of Toll-like receptor 2 (TLR2), induction of an immunoregulatory phenotype in DCs, and increased excitability of intestinal neurons.^23^ We have recently shown that MV from JB-1 contain immunomodulatory lipoteichoic acid (LTA) and are endocytosed by intestinal epithelial cells both *in vitro* in a clathrin-dependent mechanism, as well as *in vivo*.^24^ Internalized MV from some beneficial bacteria appear to be protected against intracellular destruction^23^ and may even transcytose the epithelium.^25^ This suggests the possibility that bacterial MV may circulate from the gut and additionally mediate some systemic effects associated with the parent bacterium.

Here, we show that nanoparticles isolated from the plasma of mice fed with JB-1 are TLR2-active and immunomodulatory *in vitro*. This activity is inhibited by neutralizing antibodies against LTA, while isolated particles contain labeled proteins and bacteriophage DNA of JB-1 origin. These data suggest that MV from a beneficial gram-positive bacterium in the gut circulate systemically shortly after its consumption and may drive its systemic influence.

## Materials and methods

### Animals

Male BALB/c mice (8–10 weeks old) were obtained from Charles River (Montreal, Canada) and maintained on a 12-hour light-dark cycle in a specific pathogen-free facility with *ad libitum* access to food and water. Mice were acclimatized for at least one week after arrival and were used in experiments within 4–8 weeks. All experiments involving mice followed the Canadian Council on Animal Care guidelines and were approved by the McMaster Animal Research Ethics Board.

### Bacterial culture

*Lacticaseibacillus rhamnosus* JB-1 (JB-1) was grown anaerobically in Man-Rogosa-Sharpe (MRS) medium for 24 h, then centrifuged at 4°C and 1900 × g for 30 min to pellet bacteria. Bacterial pellets were resuspended in PBS, diluted to 10^10^ JB-1/mL according to optical density, and frozen at −80°C until use. To obtain bacterial MV, supernatants from bacterial cultures were filtered, ultracentrifuged, and fluorescently labeled (where necessary) as previously described.^24^ When appropriate, JB-1 was fluorescently labeled with 20 μm CFSE (CFDA SE; Invitrogen, Burlington, Canada) for 20 min at 37°C in the dark, washed in cold PBS, then used immediately in equivalent dose to unlabeled JB-1.

### Plasma EV isolation

Mice were orally gavaged in groups of 6–12 animals with 200 μL PBS or approx. 2 × 10^9^ *L. rhamnosus* JB-1 in PBS, then 2.5 h later were exsanguinated by decapitation. Blood was collected from individual mice in EDTA-containing tubes, then sequentially centrifuged at 6000 × g and 9000 × g for 15 min at 4°C to remove cells and platelets. Plasma from individual animals of each group were then pooled, diluted in cold PBS, and ultracentrifuged at 42,000 RPM (121,000 × g) at 4°C in a Type 70 Ti fixed-angle rotor (Beckman Coulter, Mississauga, Canada) for 16 h followed by 3 h, with washing in PBS between spins. EV were resuspended at a final volume of 200 μL per contributing mouse, then aliquoted and stored at −80°C until further use. Protein concentration was measured by Pierce Rapid Gold bicinchoninic acid assay (Thermo Fisher, Mississauga, Canada). Where applicable, mice were instead gavaged with approximately 3 × 10^10^ JB-1 MV in 200 μL PBS and plasma EV collected as described above.

### Nanoparticle tracking analysis

Particle concentration and size distributions were determined by nanoparticle tracking analysis (NanoSight NS300; Malvern Panalytical, Montreal, Canada) at the Structural & Biophysical Core Facility at the Hospital for Sick Children (Toronto, Canada). EV or MV preps were diluted in sufficient PBS to measure 30–100 particles per frame, then run with a continuous flow syringe pump through a 532 nm laser. Five 60-second videos were captured and analyzed using NTA software (v. 3.2, build 3.2.16; Malvern Panalytical) with camera level 16 and detection threshold of 5.

### Transmission electron microscopy

Electron microscopy of EV samples was performed as recently described.^24^ Briefly, EV were deposited onto formvar-coated copper grids, negatively stained with 1% aqueous uranyl acetate, and viewed in a JEOL 1200 EX TEMSCAN transmission electron microscope (JEOL, Peabody, USA) operating at 80 kV. Micrographs were taken with a 4-megapixel digital camera (Advanced Microscopy Techniques, Woburn, USA).

### Cell culture

T84 cells were a gift from Dr. Ali Ashkar (McMaster University) and were cultured in a 5% CO_2_ incubator at 37°C in DMEM/F-12 with L-glutamine, HEPES, 100 U/mL penicillin, 100 μg/mL streptomycin, and 10% fetal bovine serum (FBS). HEK-Blue mTLR2 cells were obtained from InvivoGen (San Diego, USA) and cultured as per manufacturer’s directions and as recently described^24^ in supplemented DMEM. BMDCs were derived as previously described from BALB/c mouse tibia and femurs.^23,24,26^

### TLR2 assay

TLR2 ligand presence was determined using a mouse TLR2 reporter cell line (HEK-Blue-mTLR2; Invivogen) following manufacturer’s directions and as recently described.^24^ Where indicated, cells were pre-incubated with anti-LTA antibody (5 μg/mL; clone G43J, Invitrogen) for 1 h prior to sample addition. Positive control wells were incubated with the TLR2 agonist Pam3CSK4 (300 ng/mL). Standard curves of JB-1 MV were performed by serial dilution of characterized MV preparations. After sample incubation, cell-free supernatants were added to the detection reagent and incubated at 37°C for 1 h, then measured spectrophotometrically at 650 nm.

### BMDC flow cytometry analysis for IL-10 expression

BMDC IL-10 expression was assessed as previously described.^24^ Briefly, 10^6^ BMDCs were seeded per well in 6-well plates in 450 μL complete RPMI medium. If necessary, cells were pre-incubated with anti-LTA antibody (5 μg/mL) for 1 h, then incubated with 50 μL sample at 37°C for 18 h. Cells were resuspended, washed, Fc blocked, and incubated with anti-CD11c-PerCP-Cy5.5 (1:200; Invitrogen) for 30 min. Cells were fixed and permeabilized using a BD Cytofix/Cytoperm kit (BD Biosciences, San Jose, USA) and incubated with anti-IL-10-PE (1:200; Invitrogen) for 30 min. After washing, fluorescence was measured by flow cytometry in a FACSCelesta flow cytometer (BD Biosciences) and analyzed in FlowJo (v. 9.4; BD Biosciences, Ashland, USA).

### TNF-induced IL-8 expression by T84 cells

T84 cells were plated on 6-well cell culture plates at 10^6^ cells/well in 4 mL normal growth medium. Three days later, cells were washed and media replaced with total 2 mL antibiotic-free growth medium containing 200 μL EV samples or PBS. Cells were incubated with samples for 2 h, then 2.5 ng/mL recombinant human TNF (Gibco) was added as appropriate and incubated for an additional 2 h. Supernatants were collected and immediately frozen at −80°C until further processing. To measure secreted IL-8 concentrations, supernatants were thawed, centrifuged (500 × g, 7 min) to pellet debris, and analyzed by IL-8 ELISA (Invitrogen) according to manufacturer’s directions.

### Proteomics

Proteomics analyses were performed by the Center for Advanced Proteomics Analyses, a Node of the Canadian Genomic Innovation Network that is supported by the Canadian Government through Genome Canada. Briefly, samples in PBS (approx. 200 μg protein; 3 paired samples per group) were dried, reconstituted in 100 μL 50 mM ammonium bicarbonate with 10 mM TCEP-HCl, and vortexed for 1 h at 37°C. Chloroacetamide (55 mM) was added for alkylation and samples vortexed for 1 h at 37°C. Trypsin (1 μg) was then added and samples digested for 8 h at 37°C. Samples were dried and solubilized in 5% acetonitrile and 0.2% formic acid, then loaded on a 1.5 μL pre-column (Optimize Technologies, Oregon City, USA). Peptides were separated on a home-made reversed-phase column (150 μm inner diameter by 200 mm) with a 56 min gradient from 10% to 30% acetonitrile with 0.2% formic acid and a 600 nL/min flow rate on an Easy nLC-1000 connected to a Q Exactive HF Orbitrap LC-MS/MS System (Thermo Scientific, San Jose, USA). Each full MS spectrum, acquired at a resolution of 60,000, was followed by tandem-MS spectra acquisition on the 15 most abundant multiply charged precursor ions. Tandem-MS experiments were performed using higher energy collision dissociation at a collision energy of 27%. Data were processed using PEAKS X (Bioinformatics Solutions, Waterloo, Canada) and the UniProt mouse and *L. rhamnosus* GG (ATCC 53103) databases. Mass tolerances on precursor and fragment ions were 10 ppm and 0.01 Da, respectively. Fixed modification was carbamidomethyl. Variable selected posttranslational modifications were oxidation, deamidification, and phosphorylation. The data were visualized with Scaffold (v. 4.3.0; Proteome Software, Portland, USA), protein threshold 99%, with at least 2 peptides identified and a false-discovery rate of 1% for peptides). We used the average intensity of the 3 highest-intensity peptides for further analyses.

### Bioinformatics

Pairwise comparisons of all proteins detected in at least 1 sample per group were conducted by Welch’s t-test and plotted using the EnhancedVolcano package (v 1.8.0)^27^ in R (v. 3.4.4).^28^ For Gene Ontology analysis,^29,30^ proteins were filtered to include only those consistently enriched across samples (e.g., all 3 JB-1-fed EV samples higher than their paired PBS-fed EV sample) and with *p* < 0.25. Proteins that were exclusively found in all 3 samples of one group and none of the other group were also included. The resulting dataset was then split by whether proteins were upregulated or downregulated in EV from JB-1-fed mice and analyzed separately. Gene ontology enrichment analysis was performed using ShinyGO (v. 0.61).^31^ Results were converted to GO IDs using the GOfuncR package (v. 1.10.0)^32^ then reduced by removing redundant GO terms with the rrvgo package (v. 1.2.0)^33^ with a medium similarity threshold (0.7). Reduced terms were plotted with ggplot2 (v 3.3.2)^34^ as adapted from published protocols.^35^

### Fluorescence measurement

To measure CFSE fluorescence, samples were added at 50 μL per well to a black opaque 96-well plate and fluorescence measured in a SpectraMax i3x plate reader (Molecular Devices, San Jose, USA) with excitation at 492 nm and emission measured at 517 nm. Standard curves of JB-1 MV were performed by serial dilution of characterized CFSE-labeled MV preparations. Raw fluorescence values were corrected by subtracting values from PBS-containing negative control wells.

### Prophage prediction, annotation, and primer design

Predictions of PHASTER,^36^ PhiSpy,^37^ and VirSorter^38^ on the genome of *L. rhamnosus* GG (Accession: FM179322.1) were aligned to identify consensus predictions. Predictions were manually curated based on the presence of signature phage genes, such as capsid, terminase, and tail structural modules. Designated prophages were then annotated in detail. An analysis of each prophage region was conducted with ViPTree,^39^ an online server that generates a proteomic tree of viral genome sequences based on genome-wide sequence similarities computed by tBLASTx. Geneious Prime (v.2020.0.04, https://www.geneious.com) was used to design primers internal to recognizable phage genes to ensure amplification. Primers are shown in Table S7.

### Phage induction

*L. rhamnosus* JB-1 was grown aerobically to an OD_600nm_ of 0.1–0.2 in 1 L of MRS broth. Mitomycin C was added to a final concentration of 2 µg/mL and incubated for 6 h, then bacteria centrifuged at 16,300 × g for 15 min. The supernatant was collected and filtered through a 0.45 µm PES filter. Filtrates were concentrated by adding 29.2 g/mL NaCl and 100 g polyethylene glycol 8000 (PEG; final concentration of 10% w/v) and incubating overnight at 4°C with stirring. The precipitate was then centrifuged at 16,300 × g for 15 min at 4°C and the pellet resuspended in 5 mL phage buffer (50 mM Tris-HCl, pH 7.5; 100 mM NaCl; 8 mM MgSO_4_). The resulting phage concentrate was re-filtered through a 0.45 μm syringe filter and used for DNA extraction.

### Phage DNA extraction

Phage DNA was extracted from concentrates by phenol-chloroform treatment. Briefly, 1 mL of phage concentrate was incubated at 37°C for 30 min with 20 μg/mL RNAse A (New England BioLabs, 20 mg/ml stock) and 2 U/mL DNAse I (New England BioLabs) in DNAse buffer (New England BioLabs). Nucleases were inactivated with 5 mM EDTA (65°C for 10 min). Proteinase K (200 μg/mL; New England BioLabs) and 200 μL 10% SDS were then added and incubated for 1 h at 37°C to increase yield from phage capsids. Finally, one volume of phenol:chloroform:isoamyl alcohol (25:24:1) was added, vortexed, then samples centrifuged at 16,000 × g for 10 min. DNA was recovered by alcohol precipitation from the aqueous phase and resuspended in 20 μL elution buffer (10 mM Tris, 0.1 mM EDTA, pH 8.5).

### EV and endogenous bacterial DNA extraction

Nucleic acids were extracted from samples using TRIzol Reagent (Invitrogen) as per manufacturer’s directions. Samples were EV resuspended in PBS as described or cecal contents that had been collected from naïve male BALB/c mice and stored at −80°C until use. Briefly, 50 μL of samples were lysed in 450 μL TRIzol, mixed with 90 μL chloroform, and centrifuged. The aqueous phase was discarded and DNA precipitated by adding 100% ethanol to the organic phase. The pellet was washed twice in 0.1 M sodium citrate in 10% ethanol, washed once in 75% ethanol, then resuspended in 50 μL 8 mM NaOH in nuclease-free water and frozen at −20°C.

### Real-time quantitative PCR

Isolated DNA was measured by real-time qPCR using PowerUp SYBR Green Master Mix (Applied Biosystems). Standard curves of phage DNA were made by serial dilution of DNA isolated from characterized JB-1 MV preparations. Forty cycles were run with 15 sec denaturing at 95°C, 15 sec annealing at 60°C, and 1 min extending at 72°C. Primers used are shown in Table S7. PCR products were electrophoresed in a 1.5% agarose gel and visualized using a ChemiDoc Touch Imaging System (Bio-Rad, Mississauga, Canada). Full length gels are shown in Figure S5.

### Data analysis

Data were analyzed in R (v. 3.4.4)^28^ with the effsize package^40^ or others as described above. Pooled EV isolated from JB-1-fed or PBS-fed mice from the same batch (received, treated, and euthanized at the same time) were considered matched controls to reduce extraneous between-batch variability. Thus, where indicated, pairwise comparisons were done by directional t-test with Welch’s correction for unequal variances (*t*) with effect size reported as Hedge’s g (*d*). Graphs were created using the R package ggplot2^34^ or GraphPad Prism (v. 6.01) and assembled in Adobe Illustrator.

## Results

### Blood nanoparticles from JB-1-fed mice reproduce some JB-1-related activity *in* *vitro*

**Figure 1.**
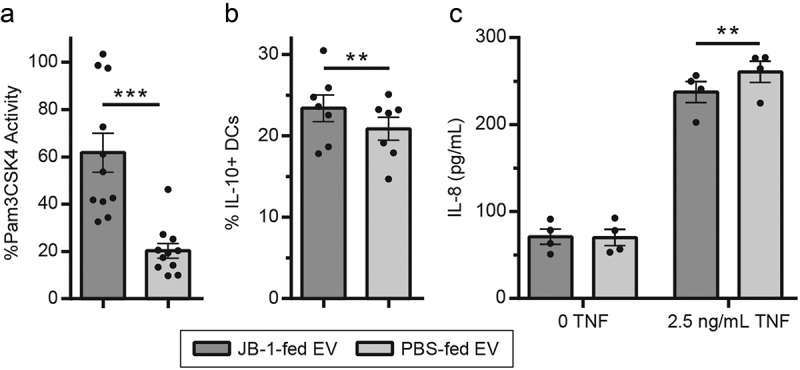
**EV from mice fed with *L. rhamnosus* JB-1 reproduce *in vitro* activity of the original bacterium**. (a) TLR2 activation by EV was quantified by colorimetric assay using a reporter cell line, with data expressed as a percentage of activity measured for the synthetic TLR2 ligand Pam3CSK4 (300 ng/mL). (b) EV were incubated with BMDCs and subsequent IL-10 expression was measured by flow cytometry. (c) EV were preincubated with T84 cells for 2 h, exposed to 0 or 2.5 ng/mL TNF for 2 h, then IL-8 secretion was measured by ELISA. Error bars represent ± 1 standard error. Each point represents one EV preparation (6–12 mice pooled per preparation). ** *p* < 0.01, ***p < 0.001

We hypothesized that effects seen in mice after consumption of JB-1 are mediated by circulating nanoparticles. To test this, we gavaged BALB/c mice with 2 × 10^9^ JB-1 bacteria or phosphate-buffered saline (PBS) vehicle, then 2.5 h later (coincident with JB-1-induced regional brain activation^9^) collected plasma, pooled from 6–12 animals per group, and ultracentrifuged it to obtain circulating nanoparticles (which for simplicity we refer to as EV). We then tested EV for effects seen with JB-1 treatment *in vitro*.^5,23,41^ Using a TLR2 reporter cell line, we found that EV from mice fed with JB-1 activated TLR2 to a far greater extent than did those from mice fed with PBS (*t_10_* = 5.69, *d* = 1.78, *p* < 0.0001; Figure 1a). When incubated with bone marrow-derived dendritic cells (BMDCs), these EV induced higher IL-10 expression (*t_6_* = 3.2, *d* = 0.60, *p* = 0.0094; Figure 1b). And in a model of TNF-induced IL-8 expression in a human intestinal epithelial cell line (T84), EV from JB-1-fed mice inhibited IL-8 release to a greater extent than did those from PBS-fed mice (*t_3_* = −5.11, *d* = −0.96, *p* = 0.007; Figure 1c). These results demonstrate that nanoparticles with immune-modulating activity analogous to JB-1 are present in plasma of mice shortly after consumption of this bacterium.

### Neuronal proteins are relatively enriched in blood nanoparticles from JB-1-fed mice

**Figure 2.**
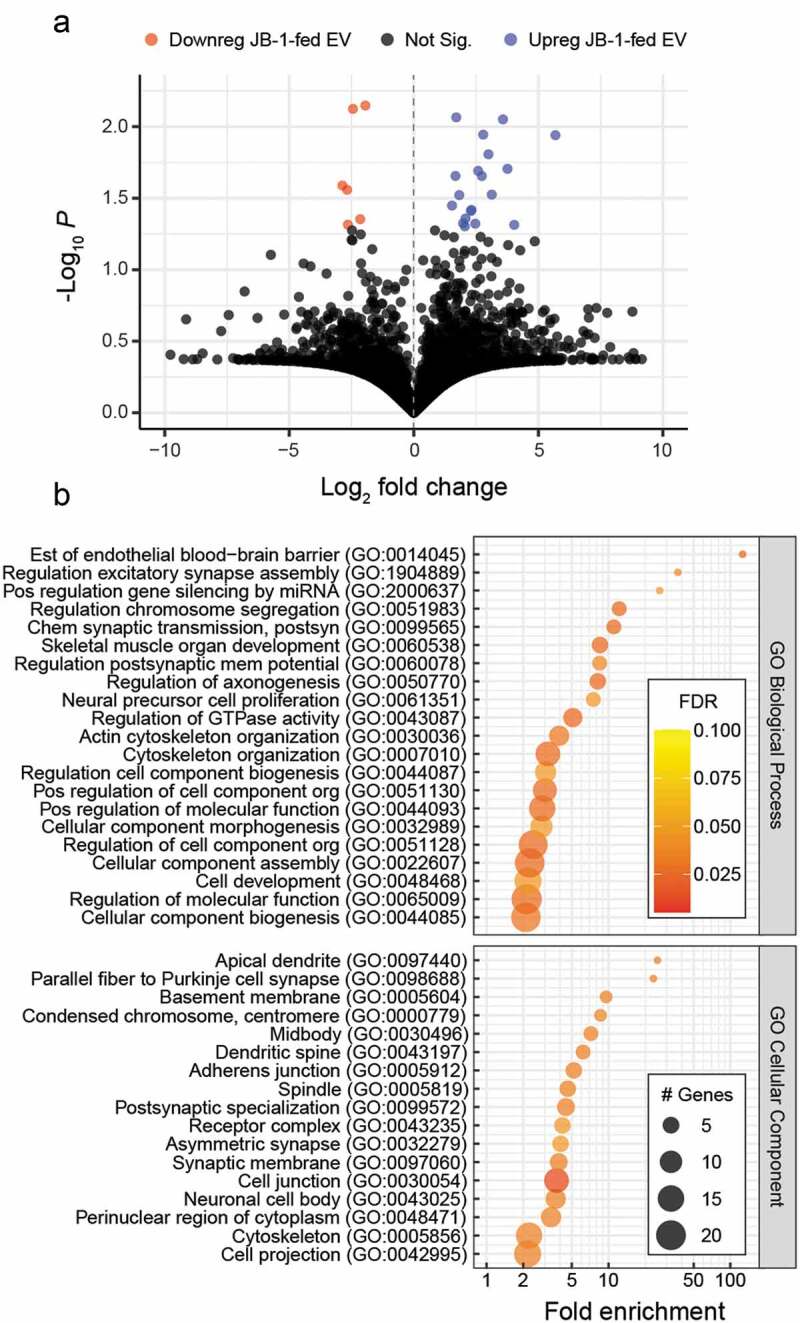
**EV from *L. rhamnosus* JB-1-fed mice are enriched in neuronal proteins**. (a) Volcano plot comparing relative intensities of individual proteins detected in EV from JB-1- or PBS-fed mice. (b) Gene Ontology enrichment analysis of EV proteins that were enriched in EV from JB-1-fed mice relative to those of PBS-fed mice, showing terms with FDR < 0.05. Data are from 3 EV preparations per group

To investigate whether JB-1 consumption influenced the mouse plasma EV proteome, we performed proteomic analyses using the UniProt mouse database. We detected 3421 proteins between JB-1- and PBS-fed EV samples. Of these, 19 were significantly increased in EV from JB-1-fed mice, while 6 were significantly decreased (Figure 2a; Table S1). In addition, we found 19 proteins that were exclusively detected in all EV preps from JB-1-fed mice, and 17 that were exclusively detected in those from PBS-fed mice (Table S2). We used Gene Ontology^29,30^ enrichment analysis to determine whether enriched proteins related to any particular processes. Interestingly, proteins upregulated in EV from JB-1-fed mice were enriched in terms related to neuronal structure and function, along with terms associated with more general cellular processes (Figure 2b; Table S3). Analysis of proteins downregulated in EV from JB-1-fed mice did not reveal any significantly enriched terms. None of the identified proteins or pathways have any identifiable immune activity or are associated with known immune pathways relevant to the functions we assessed. Instead, they may reflect EV released during increased peripheral and central neuronal activity known to be induced by JB-1 consumption.^7,9^

### Consumption of JB-1 influences the composition of circulating mouse nanoparticles

**Figure 3.**
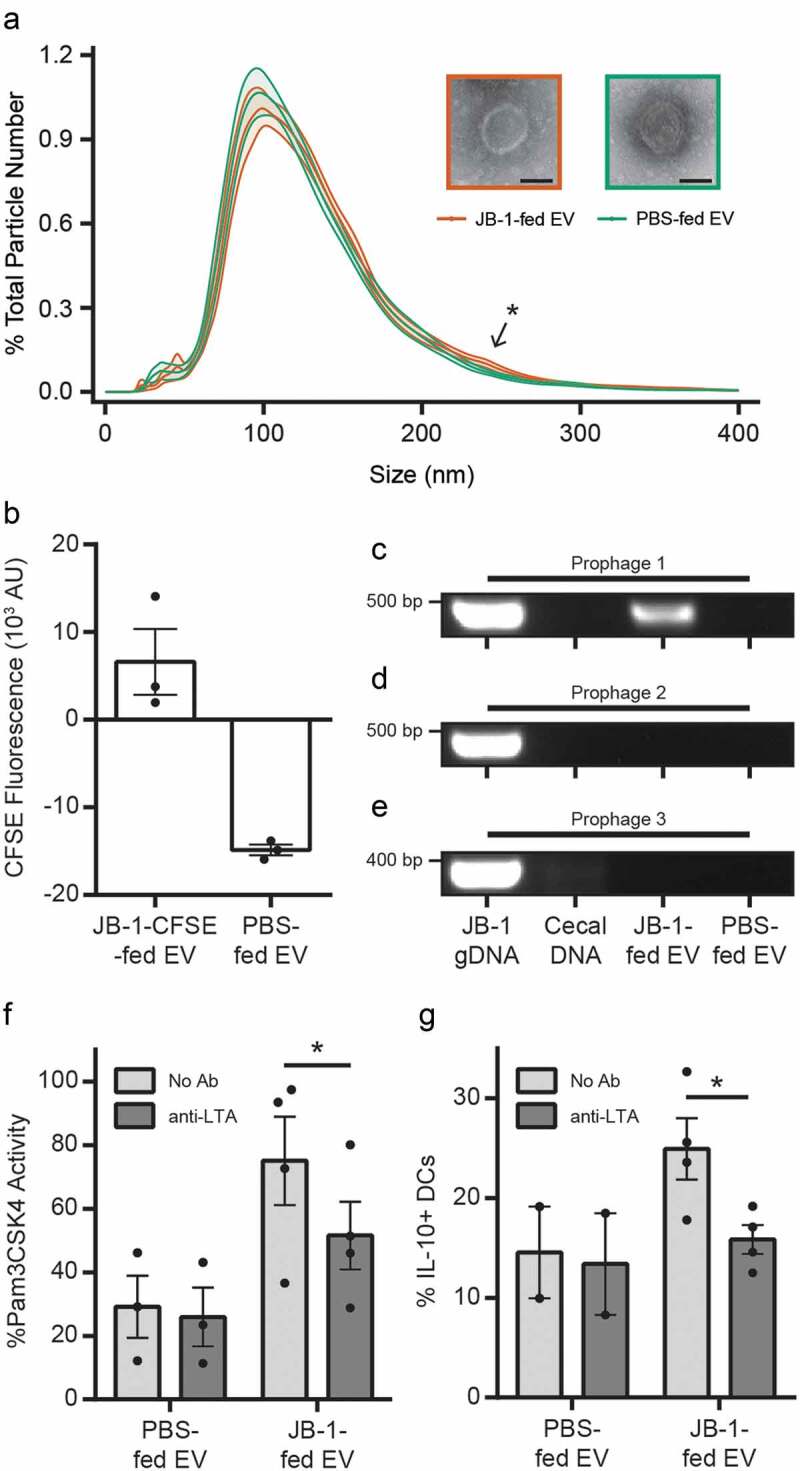
**EV isolated from plasma of mice fed *L. rhamnosus* JB-1 have a distinctive size distribution and contain protein, phage DNA, and lipoteichoic acid of JB-1 origin**. (a) Nanoparticle tracking analysis (graph) was used to characterize the size distribution of EV from mice fed JB-1 or PBS vehicle, while transmission electron microscopy (inset images) was used to visualize them. Scale bars represent 50 nm. Ribbon represents ± 1 standard error of 12 EV preparations. Arrow and asterisk indicate region of significant differences (*p* < 0.05). (b) Plasma EV from mice fed with CFSE-labeled JB-1 or PBS vehicle were assessed for CFSE-related fluorescence using a plate reader. Data are shown after subtraction of PBS blank wells. (c-e) DNA electrophoresis of qPCR products showing (c) Prophage 1 DNA detected in JB-1 genomic DNA (gDNA) and EV from JB-1-fed mice, but not naïve mouse cecal contents nor EV from PBS-fed mice, whereas (d) Prophage 2 and (e) Prophage 3 were detected only in JB-1 genomic DNA. Full-length gels shown in Fig. S5. (f) EV were assessed for TLR2 activity in a reporter cell line after pre-incubation with or without anti-LTA antibody. Data are expressed as a percentage of activity measured for the synthetic TLR2 ligand Pam3CSK4 (300 ng/mL). (g) BMDCs expressing IL-10 were counted by flow cytometry after pre-incubation with or without anti-LTA antibody and incubation with EV samples. Error bars represent ± 1 standard error. * *p* < 0.05

By nanoparticle tracking analysis (Figure 3a) we found that EV from JB-1-fed mice had a mean diameter of 134 nm (mode 105 nm), while those from PBS-fed mice had a mean diameter of 130 nm (mode 103 nm), though this was not a statistically significant difference (*t*_11_ = 1.73, *d* = 0.34, *p* = 0.11). Nor was the difference in average concentrations of EV significant between groups (*t*_11_ = 0.97, *d* = 0.15, *p* = 0.35), with an average of 2.6 × 10^10^ EV/mL from JB-1-fed mice and 2.3 × 10^10^ EV/mL from PBS-fed mice. An analysis of variance to compare normalized size distributions between EV from JB-1- and PBS-fed mice revealed a significant group by size interaction (*F* = 9.3, *η*^2^*_G_* = 0.002, *p* = 0.002), while follow-up pairwise comparisons revealed significant differences (*p*s < 0.05) from 234 nm to 254 nm (*d*s = 0.51– 0.61) as indicated in Figure 3a. These data suggest that feeding with JB-1 influences the composition of circulating EV.

### Blood nanoparticles contain protein from fed bacteria

To test whether EV from JB-1-fed mice contain protein of JB-1 origin, we labeled JB-1 with carboxyfluorescein succinimidyl ester (CFSE), a fluorophore that covalently and stably binds intracellular amines that we have previously used to label JB-1 MV.^23,24^ We confirmed that CFSE did not impair bacterial growth (Fig. S1), then gavaged CFSE-labeled JB-1 to mice and collected plasma EV 2.5 h later as usual. Using a fluorescent plate reader, we found detectable fluorescence in EV preparations from CFSE-labeled JB-1-fed mice (Figure 3b).

### *L.* *rhamnosus*JB-1 encodes inducible prophages

The presence of CFSE-labeled protein led us to wonder whether bacterial proteins were detectable in EV from JB-1-fed mice. We thus re-analyzed our proteomics data using the database for another well-characterized related strain, *L. rhamnosus* GG (accession: FM179322.1). While 95 proteins were detected across both groups, we suspect most were the result of homology with mitochondrial proteins or bacterial proteins from the indigenous microbiota, as only four were absent in all PBS-fed EV preparations. Of those, only two were detected in at least two of the three JB-1-fed EV preparations: a predicted phosphoglucosamine mutase (LGG_00981) and a predicted phage tail related component (LGG_01524). The latter caused us to investigate whether JB-1 might be carrying a functional bacterial virus (phage) integrated in its genome in a dormant state (prophage), which could also be a component of the EV preparations.

Leveraging three prophage prediction tools on the genome of *L. rhamnosus* GG we predicted the existence of three intact prophages (Fig. S2), potentially capable of induction (awakening), lysing the host bacterium, and releasing phage progeny. Prophages 1 (~42 kb; Table S4) and 2 (~33 kb; Table S5) share clear homology with portions of bacteriophage iLp84 (NC_028783) and Lactobacillus phage Lc-Nu (NC_007501), respectively. In contrast, Prophage 3 is much smaller (~15 kb; Table S6), with no close phage homologues. According to an analysis by ViPTree,^39^ all 3 cluster with viruses of the family *Siphoviridae*.

To validate those predictions we performed classical phage induction experiments. We exposed *L. rhamnosus* JB-1 to the DNA-damaging agent Mitomycin C, filtered out bacterial components, treated with DNAse to remove any free-floating DNA, and used primers specific to each of these phage regions to detect the presence of protein-protected phage DNA, consistent with a functional phage particle. All three predicted prophages were detectable in extractions from concentrated filtrates (Fig. S3A), while bacterial 16S was not (Fig. S3B), confirming that they are intact phages that can exist as particles independently of the JB-1 bacterium.

To determine whether these phages naturally associate with cultured JB-1 MV in the absence of stressors, we applied an identical DNAse-treatment followed by PCR to detect phage DNA within JB-1 MV preparations. While DNA from all three phages was detectable even after DNAse treatment, only Prophage 1 was consistently detectable at a high level (Fig. S4).

### Blood nanoparticles from JB-1-fed mice contain bacteriophage DNA

The strong association of Prophage 1 with JB-1 MV in culture suggested that its DNA could serve as a barcode for the presence of JB-1-origin nanoparticles in blood EV from JB-1-fed mice. We first tested whether any of these three phages were detectable in DNA isolated from naïve mouse microbiota. We did not convincingly detect DNA from Prophages 1, 2, or 3 in cecal contents (Figure 3c-e), although the primers for Prophage 3 did amplify faint products across a broad size range, suggesting some nonspecific binding (Fig. S5). We then examined whether phage DNA was detectable in blood EV samples by qPCR. We found Prophage 1 DNA in JB-1-fed EV preparations (average 33 cycles) but not those from PBS-fed mice (Figure 3c). We did not detect DNA from Prophages 2 or 3 (Figure 3d-e), suggesting an enrichment in replicated Prophage 1 DNA and not simply chromosomal DNA, where all three phages would be equally represented. This likely reflects the presence of Prophage 1 particles in the blood EV preparations, although it is possible the replicative phage is an abundant replicon in the cells, and therefore its DNA more likely to be found in MV including intracellular content. Regardless, these data show that nucleic acids from JB-1 are present in nanoparticulate isolates from the blood of mice fed with JB-1.

### Activity of nanoparticles from blood of JB-1-fed mice is mediated by lipoteichoic acid

We have recently shown that JB-1 MV contain immunomodulatory LTA.^24^ To determine whether the same activities are present in EV from JB-1-fed mice, we used antibody neutralization experiments. Preincubation with anti-LTA antibodies significantly decreased the effect of EV from JB-1- but not PBS- fed mice on both TLR2 activation in a reporter cell line (*t*_3_ = −3.22, *d* = −0.86, *p* = 0.024; figure 3f) and IL-10 expression by BMDCs (*t_3_* = −3.00, *d* = 1.81, *p* = 0.029; Figure 3g). Altogether, these experiments show that bacterial LTA is present in EV isolated from JB-1-fed mice and largely mediates their immune activity.

### Estimated number of bacteria-derived particles in plasma

**Figure 4.**
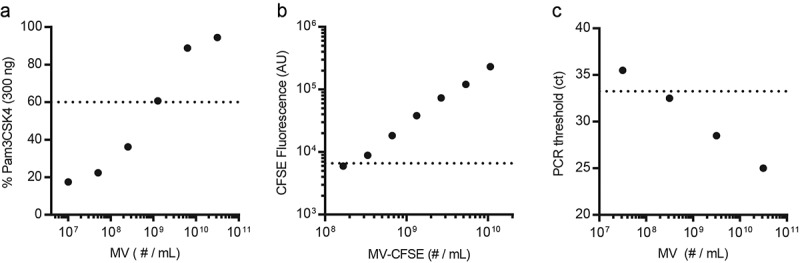
**Estimation of bacterial MV in plasma EV isolated from mice fed *L. rhamnosus* JB-1**. We enumerated JB-1 MV by nanoparticle tracking analysis and used these to produce standard curves for (a) MV activation of TLR2 in a reporter cell line, (b) CFSE fluorescence after labeling MV with CFSE, and (c) qPCR cycle number at which Prophage 1 DNA was detected (ct). Horizontal dotted lines show the average values of EV from JB-1-fed mice in the same assays. Where the horizontal line and trend of response intersect is an estimate of the number of MV in an average EV preparation

The presence of bacterial protein, DNA, and LTA from the original JB-1 inoculum in EV from JB-1-fed mice strongly suggests the presence of whole bacterial MV in these isolates. To estimate the relative size of this population of nanoparticles, we compared JB-1-fed EV preparations to standard curves of characterized JB-1 MV from pure bacterial culture. By combining these three independent methods we estimate that between 10^8^ to 10^9^ MV/mL are present in the plasma of mice fed with JB-1 (Figure 4a-c). Given an average of 2.6 × 10^10^ EV/mL measured by nanoparticle tracking analysis described above, this suggests that 0.4%–4% of nanoparticles collected from plasma of JB-1-fed mice are bacterial in origin.

### JB-1 MV preparations appear in blood after oral consumption

Our data suggest that JB-1 MV produced *in situ* in the gut translocate the epithelium and enter the blood. We wondered whether similar translocation would be seen after feeding JB-1 MV directly. We thus labeled JB-1 MV with CFSE, gavaged them to BALB/c mice, and collected EV as before. These EV contained CFSE fluorescence and activated TLR2, albeit to a lesser extent than seen after feeding JB-1 bacteria (Fig. S6A-B). Interestingly, DNA isolated from EV of JB-1 MV-fed mice contained both Prophage 1 and Prophage 3 DNA (Fig. S6C), consistent with the phages released by JB-1 under normal culture conditions (Fig. S4).

## Discussion

Bacterial MV are promising mediators of bacteria-host communication, but whether this is confined to the intestinal lumen or relevant to systemic influence is unclear. Here we show that within 2.5 h of consumption of the bacterium *L. rhamnosus* JB-1 by mice there are functional EV circulating in blood that contain activity associated with the fed bacterium. These EV appear to be bacterial MV at least in part as their activity was inhibited by anti-LTA antibodies and the particles contained both fluorescent protein and phages of JB-1 origin.

We have previously shown that JB-1 inhibits TNF-induced IL-8 release by gut epithelial cells,^41^ activates TLR2,^23^ and induces an immunoregulatory phenotype in DCs,^5^ all of which also associated here with EV from blood of JB-1-fed but not PBS-fed mice. Moreover, we recently demonstrated that JB-1 MV contain LTA, which activates TLR2 and induces IL-10 production by DCs *in vitro*.^24^ That EV-related activity here was inhibited by anti-LTA antibody supports that these effects are largely mediated by nanoparticle-associated JB-1 LTA. Moreover, these data suggest that bioactive surface components of a probiotic bacterium are not only present on circulating nanoparticles *in vivo*, but also retain activity despite passage through the gastrointestinal tract. This may have important implications for therapeutic applications of such bacteria, which could be mediated by structurally variable components such as LTA.^24^

We examined plasma nanoparticles 2.5 h after feeding JB-1 because we have recently shown that JB-1 produces regional brain activation within this time period.^9^ This interval is also consistent with findings of Karlsson and colleagues, who described the production of blood nanoparticles (termed *tolerosomes*) by intestinal epithelial cells within 2 h of consumption of a large dose of ovalbumin,^42^ which mediated antigen-specific tolerance in an MHC II-dependent manner.^43^ Our estimate that between 0.4%-4% of the particles isolated from the blood of mice fed with JB-1 are bacterial in origin may be imperfect, but nonetheless is within the range of concentration differences between JB-1-fed and PBS-fed EV as measured by nanoparticle tracking analysis. And though we did detect some low-level activity in PBS-fed mouse blood, presumably from indigenous gut bacteria, our estimate does not ignore this baseline. Since JB-1 MV can be rapidly endocytosed by mouse intestinal epithelial cells,^24^ our data support a mechanism by which LTA-containing MV are rapidly internalized by gut epithelial cells, transcytose, and circulate systemically, thereby influencing systems distal from the gut similar to the effects of the parent JB-1 bacteria.^4–6^

In support of this, Rubio and colleagues recently reported that *Bacillus subtilis* MV are rapidly transcytosed by the human colonic epithelial cell line Caco-2,^25^ while Tulkens and colleagues demonstrated that lipopolysaccharide from gram-negative MV is detectable in human blood, especially in individuals with intestinal barrier dysfunction.^44^ In mice, consumption of *Bacteroides thetaiotaomicron* OMV labeled with a lipid-soluble fluorophore resulted in fluorescence detectable in various organs within 8 hours, most notably in the liver.^45^ And from a functional perspective, Aoki-Yoshida and colleagues demonstrated that nanoparticles precipitated from blood of mice fed with *Lactobacilli* for 7 days had anti-inflammatory effects on phagocytes *in vitro*.^46^ While the authors inferred that these effects were due to immunomodulatory exosomes in circulation, we suggest that they may also have been driven by the mechanism we propose here.

We did not find evidence to suggest that host EV are involved in the nanoparticulate effects seen in this study. Proteomic analyses did not indicate any functionally relevant mouse proteins, though we did see an enrichment in proteins related to neuronal physiology. This likely reflects the well-established activities of both JB-1 and its MV on central and peripheral nervous system function^7,23,47–49^ resulting in heightened release of neuronal EV into circulation (such as from the enteric nervous system).

To further characterize that active blood particles were bacterial in origin, we fluorescently labeled *L. rhamnosus* JB-1 with CFSE, which stably binds to intracellular primary amines and fluoresces for prolonged time periods *in vivo*.^50^ Thus, the fluorescence detected in EV from mice fed with labeled JB-1 reflects cytosolic JB-1 proteins that were likely packaged into MV *in situ*, consistent with our findings after gavage of directly labeled MV. Moreover, that EV from mice fed with JB-1 or its MV contain components of JB-1 itself is confirmed by our detection of bacteriophage DNA found uniquely in JB-1 and not in the indigenous cecal microbiota. While at this stage we cannot be certain in what form this phage DNA was present in the blood, we propose that JB-1 in the gut lumen produces MV that contain phage DNA and transcytose into the circulation. Indeed, previous work has shown that phage nucleic acid^51^ and even entire phages^52^ can be contained within bacterial MV. Since phage-encoded endolysins are thought to be involved in production of MV by some gram-positive bacteria,^53^ MV production should be increased during active phage replication, suggesting that MV may be enriched for phage DNA.

Nonetheless, it is also possible that the phage DNA detected represents an independent set of particles in circulation. Phages can be endocytosed by gut epithelial cells and remain viable for prolonged periods^54^ and they may also transcytose the gut epithelium.^55^ Active gut bacterial phages can be found in blood samples from healthy individuals.^56,57^ Moreover, phages may directly modulate mammalian systems.^58^ Recent work has shown that phages, despite being unable to replicate within mammalian cells, can nonetheless modulate host interferon responses.^59,60^ Importantly, our use of CFSE could also have labeled phage proteins in bacteria resulting in labeled phage in blood samples. While some viral capsids have been shown to activate TLR2,^61^ it seems unlikely that anti-LTA would inhibit TLR2-activating phage protein. We do not know whether the identified nanoparticulate phage DNA represents active phages, but if this occurs with other beneficial strains or probiotics then it may represent an additional novel pathway whereby such bacteria can have systemic effects.

In summary, we have shown that oral consumption of a beneficial bacterium leads to circulating nanoparticles containing bacterial components that are likely bacteria-produced membrane vesicles. That these particles were active in multiple systems and reproduced activity associated with the parent bacterium supports the possibility that circulating bacterial MV can mediate their systemic influence. Our data further suggest that gut bacterial phages may be involved in their systemic communication and opens the door to further study of the role of MV and phages in host-bacterial communication and probiotic efficacy.

## Supplementary Material

Supplemental Material

## Data Availability

The data supporting the findings of this study are available within the article or its supplementary materials or are available from the authors upon request.

## References

[1] Forsythe P, Kunze W, Bienenstock J Moody microbes or fecal phrenology: what do we know about the microbiota-gut-brain axis? BMC Med [Internet]; 2016 [cited 2016 Sep 12]; 14. Available from: http://bmcmedicine.biomedcentral.com/articles/10.1186/s12916-016-0604-8 PMC4836158 27090095

[2] Schroeder BO, Bäckhed F. Signals from the gut microbiota to distant organs in physiology and disease. Nat Med. 2016;22(10):1079–15. doi:10.1038/nm.4185. 27711063

[3] Neuman H, Debelius JW, Knight R, Koren O. Microbial endocrinology: the interplay between the microbiota and the endocrine system. FEMS Microbiol Rev. 2015;39:509–521. doi:10.1093/femsre/fuu010. 25701044

[4] Karimi K, Inman MD, Bienenstock J, Forsythe P. *Lactobacillus reuteri* –induced regulatory T cells protect against an allergic airway response in mice. Am J Respir Crit Care Med. 2009;179(3):186–193. doi:10.1164/rccm.200806-951OC. 19029003

[5] Karimi K, Kandiah N, Chau J, Bienenstock J, Forsythe P. A *lactobacillus rhamnosus* strain induces a heme oxygenase dependent increase in Foxp3+ regulatory T cells. PLoS ONE. 2012;7:e47556. doi:10.1371/journal.pone.0047556. 23077634 PMC3471882

[6] Forsythe P, Wang B, Khambati I, Kunze WA. Systemic effects of ingested *lactobacillus rhamnosus*: inhibition of mast cell membrane potassium (IKCa) current and degranulation. PLoS ONE. 2012;7:e41234. doi:10.1371/journal.pone.0041234. 22815978 PMC3398942

[7] Bravo JA, Forsythe P, Chew MV, Escaravage E, Savignac HM, Dinan TG, Bienenstock J, Cryan JF. Ingestion of *lactobacillus* strain regulates emotional behavior and central GABA receptor expression in a mouse via the vagus nerve. Proc Natl Acad Sci. 2011;108(38):16050–16055. doi:10.1073/pnas.1102999108. 21876150 PMC3179073

[8] Liu Y, Mian MF, K-a MN, Forsythe P. CD4+CD25+ T cells are essential for behavioral effects of *lactobacillus rhamnosus* JB-1 in male BALB/c mice. Brain Behav Immun. 2020;88:451–460. doi:10.1016/j.bbi.2020.04.014. 32276029

[9] Bharwani A, West C, Champagne-Jorgensen K, K-a MN, Ruberto J, Kunze WA, Bienenstock J, Forsythe P. The vagus nerve is necessary for the rapid and widespread neuronal activation in the brain following oral administration of psychoactive bacteria. Neuropharmacology. 2020;170:108067. doi:10.1016/j.neuropharm.2020.108067. 32224131

[10] Brown L, Wolf JM, Prados-Rosales R, Casadevall A. Through the wall: extracellular vesicles in gram-positive bacteria, mycobacteria and fungi. Nat Rev Microbiol. 2015;13(10):620–630. doi:10.1038/nrmicro3480. 26324094 PMC4860279

[11] Théry C, Witwer KW, Aikawa E, Alcaraz MJ, Anderson JD, Andriantsitohaina R, Antoniou A, Arab T, Archer F, Atkin-Smith GK. Minimal information for studies of extracellular vesicles 2018 (MISEV2018): a position statement of the international society for extracellular vesicles and update of the MISEV2014 guidelines. J Extracell Vesicles. 2018;7:1535750. 30637094 10.1080/20013078.2018.1535750 PMC6322352

[12] Caruana JC, Walper SA. Bacterial membrane vesicles as mediators of microbe – microbe and microbe – host community interactions. Front Microbiol. 2020;11:432. doi:10.3389/fmicb.2020.00432. 32265873 PMC7105600

[13] Bomberger JM, MacEachran DP, Coutermarsh BA, Ye S, O’Toole GA, Stanton BA Long-distance delivery of bacterial virulence factors by *pseudomonas aeruginosa* outer membrane vesicles. PLoS Pathog [Internet]; 2009 [cited 2018 Aug 17]; 5. Available from: https://www.ncbi.nlm.nih.gov/pmc/articles/PMC2661024/10.1371/journal.ppat.1000382 PMC2661024 19360133

[14] Codemo M, Muschiol S, Iovino F, Nannapaneni P, Plant L, Wai SN, Henriques-Normark B Immunomodulatory effects of *pneumococcal* extracellular vesicles on cellular and humoral host defenses. mBio [Internet]; 2018 [cited 2020 Jan 24]; 9. Available from: http://mbio.asm.org/content/9/2/e00559-18 10.1128/mBio.00559-18 PMC5893880 29636428

[15] Furuta N, Takeuchi H, Amano A. Entry of *porphyromonas gingivalis* outer membrane vesicles into epithelial cells causes cellular functional impairment. Infect Immun. 2009;77(11):4761–4770. doi:10.1128/IAI.00841-09. 19737899 PMC2772519

[16] Johnston EL, Kufer TA, Kaparakis-Liaskos M. Immunodetection and pathogenesis mediated by bacterial membrane vesicles [Internet]. In: Kaparakis-Liaskos M, Kufer TA, editors. Bacterial Membrane Vesicles: biogenesis, Functions and Applications. Cham: Springer International Publishing; 2020. p. 159–188. cited 2021 Feb 12]. Available from: 10.1007/978-3-030-36331-4_8.

[17] Choi Y, Kwon Y, Kim D-K, Jeon J, Jang SC, Wang T, Ban M, Kim M-H, Jeon SG, Kim M-S. Gut microbe-derived extracellular vesicles induce insulin resistance, thereby impairing glucose metabolism in skeletal muscle. Sci Rep [Internet]; 2015 [cited 2018 Feb 10]; 5. Available from: https://www.ncbi.nlm.nih.gov/pmc/articles/PMC4625370/10.1038/srep15878 PMC4625370 26510393

[18] M-j F, Rodríguez-Nogales A, Garrido-Mesa J, Algieri F, Badía J, Giménez R, Gálvez J, Baldomà L Intestinal anti-inflammatory effects of outer membrane vesicles from *escherichia coli* nissle 1917 in DSS-experimental colitis in mice. Front Microbiol [Internet]; 2017 [cited 2018 May 21]; 8. Available from: https://www.ncbi.nlm.nih.gov/pmc/articles/PMC5504144/10.3389/fmicb.2017.01274 PMC5504144 28744268

[19] López P, González-Rodríguez I, Sánchez B, Gueimonde M, Margolles A, Suárez A. Treg-inducing membrane vesicles from *bifidobacterium bifidum* LMG13195 as potential adjuvants in immunotherapy. Vaccine. 2012;30(5):825–829. doi:10.1016/j.vaccine.2011.11.115. 22172507

[20] Shen Y, Giardino Torchia ML, Lawson GW, Karp CL, Ashwell JD, Mazmanian SK. Outer membrane vesicles of a human commensal mediate immune regulation and disease protection. Cell Host Microbe. 2012;12(4):509–520. doi:10.1016/j.chom.2012.08.004. 22999859 PMC3895402

[21] Alvarez C-S, Badia J, Bosch M, Giménez R, Baldomà L Outer membrane vesicles and soluble factors released by probiotic *escherichia coli* nissle 1917 and commensal ecor63 enhance barrier function by regulating expression of tight junction proteins in intestinal epithelial cells. Front Microbiol [Internet]; 2016 [cited 2018 Apr 16]; 7. Available from: https://www.ncbi.nlm.nih.gov/pmc/articles/PMC5156689/10.3389/fmicb.2016.01981 PMC5156689 28018313

[22] Badia J, Baldomà L. Membrane vesicles from the gut microbiota and their interactions with the host [Internet]. In: Kaparakis-Liaskos M, Kufer TA, editors. Bacterial Membrane Vesicles: biogenesis, Functions and Applications. Cham: Springer International Publishing; 2020. p. 189–217. cited 2021 Feb 12]. Available from: 10.1007/978-3-030-36331-4_9.

[23] Al-Nedawi K, Mian MF, Hossain N, Karimi K, Mao Y-K, Forsythe P, Min KK, Stanisz AM, Kunze WA, Bienenstock J. Gut commensal microvesicles reproduce parent bacterial signals to host immune and enteric nervous systems. FASEB J. 2015;29:684–695. doi:10.1096/fj.14-259721. 25392266

[24] Champagne-Jorgensen K, Mian MF, K-a MN, Stanisz AM, Bienenstock J. Membrane vesicles of lacticaseibacillus rhamnosus JB-1 contain immunomodulatory lipoteichoic acid and are endocytosed by intestinal epithelial cells. Sci Rep. 2021;11:13756. doi:10.1038/s41598-021-93311-8. 34215822 PMC8253831

[25] Rubio APD, Martínez J, Palavecino M, Fuentes F, CMS L, Marcilla A, Pérez OE, Piuri M. Transcytosis of *bacillus subtilis* extracellular vesicles through an in vitro intestinal epithelial cell model. Sci Rep. 2020;10:3120. doi:10.1038/s41598-020-60077-4. 32080346 PMC7033168

[26] Lutz MB, Kukutsch N, Ogilvie ALJ, Rößner S, Koch F, Romani N, Schuler G. An advanced culture method for generating large quantities of highly pure dendritic cells from mouse bone marrow. J Immunol Methods. 1999;223(1):77–92. doi:10.1016/S0022-1759(98)00204-X. 10037236

[27] Blighe K, Rana S, Turkes E, Ostendorf B, Lewis M. EnhancedVolcano: publication-ready volcano plots with enhanced colouring and labeling [internet]. Bioconductor version: Release (3.12); 2021 [cited 2021 Apr 5]. Available from: https://bioconductor.org/packages/EnhancedVolcano/

[28] R Core Team. R: a language and environment for statistical computing [internet]. Vienna, Austria: R Foundation for Statistical Computing; 2018. [accessed 2019 Feb 06]. Available from: https://www.R-project.org/

[29] Ashburner M, Ball CA, Blake JA, Botstein D, Butler H, Cherry JM, Davis AP, Dolinski K, Dwight SS, Eppig JT. Gene ontology: tool for the unification of biology. The gene ontology consortium. Nat Genet. 2000;25(1):25–29. doi:10.1038/75556. 10802651 PMC3037419

[30] Gene Ontology Consortium. The gene ontology resource: enriching a GOld mine. Nucleic Acids Res. 2021;49:D325–34. doi:10.1093/nar/gkaa1113. 33290552 PMC7779012

[31] Ge SX, Jung D, Yao R. ShinyGO: a graphical gene-set enrichment tool for animals and plants. Bioinformatics. 2020;36:2628–2629. doi:10.1093/bioinformatics/btz931. 31882993 PMC7178415

[32] Grote S GOfuncR: gene ontology enrichment using FUNC [internet]. 2020 [cited 2021 Feb 10]. Available from: https://bioconductor.org/packages/GOfuncR/

[33] Sayols S rrvgo: a bioconductor package to reduce and visualize gene ontology terms [internet]. Bioconductor version: Release (3.12); 2020 [cited 2021 Feb 10]. Available from: https://bioconductor.org/packages/rrvgo/

[34] Wickham H. ggplot2: elegant Graphics for Data Analysis. New York, USA: Springer-Verlag; 2016.

[35] Bonnot T, Gillard M, Nagel D A simple protocol for informative visualization of enriched gene ontology terms. Bio-Protoc [Internet]; 2019 [cited 2021 Feb 10]; 9. Available from: https://bio-protocol.org/e3429

[36] Arndt D, Grant JR, Marcu A, Sajed T, Pon A, Liang Y, Wishart DS. PHASTER: a better, faster version of the PHAST phage search tool. Nucleic Acids Res. 2016;44(W1):W16–21. doi:10.1093/nar/gkw387. 27141966 PMC4987931

[37] Akhter S, Aziz RK, Edwards RA. PhiSpy: a novel algorithm for finding prophages in bacterial genomes that combines similarity- and composition-based strategies. Nucleic Acids Res. 2012;40(16):e126. doi:10.1093/nar/gks406. 22584627 PMC3439882

[38] Roux S, Enault F, Hurwitz BL, Sullivan MB. VirSorter: mining viral signal from microbial genomic data. PeerJ. 2015;3:e985. doi:10.7717/peerj.985. 26038737 PMC4451026

[39] Nishimura Y, Yoshida T, Kuronishi M, Uehara H, Ogata H, Goto S. ViPTree: the viral proteomic tree server. Bioinforma Oxf Engl. 2017;33:2379–2380. doi:10.1093/bioinformatics/btx157. 28379287

[40] Torchiano M effsize: efficient effect size computation [internet]. 2017. [accessed 2019 Feb 06]. Available from: https://CRAN.R-project.org/package=effsize

[41] Ma D, Forsythe P, Bienenstock J. Live *lactobacillus reuteri* is essential for the inhibitory effect on tumor necrosis factor alpha-induced interleukin-8 expression. Infect Immun. 2004;72(9):5308–5314. doi:10.1128/IAI.72.9.5308-5314.2004. 15322027 PMC517478

[42] Karlsson M, Lundin S, Dahlgren U, Kahu H, Pettersson I, Telemo E. “Tolerosomes” are produced by intestinal epithelial cells. Eur J Immunol. 2001;31:2892–2900. doi:10.1002/1521-4141(2001010)31:10<2892::AID-IMMU2892>3.0.CO;2-I. 11592064

[43] Östman S, Taube M, Telemo E. Tolerosome-induced oral tolerance is MHC dependent. Immunology. 2005;116:464–476. 16313360 10.1111/j.1365-2567.2005.02245.x PMC1802439

[44] Tulkens J, Vergauwen G, Van Deun J, Geeurickx E, Dhondt B, Lippens L, M-a DS, Miinalainen I, Rappu P, De Geest BG. Increased levels of systemic LPS-positive bacterial extracellular vesicles in patients with intestinal barrier dysfunction. Gut. 2020;69:191–193. doi:10.1136/gutjnl-2018-317726. 30518529 PMC6943244

[45] Jones EJ, Booth C, Fonseca S, Parker A, Cross K, Miquel-Clopés A, Hautefort I, Mayer U, Wileman T, Stentz R. The uptake, trafficking, and biodistribution of *bacteroides thetaiotaomicron* generated outer membrane vesicles. Front Microbiol. 2020;11:57. doi:10.3389/fmicb.2020.00057. 32117106 PMC7015872

[46] Aoki-Yoshida A, Saito S, Tsuruta T, Ohsumi A, Tsunoda H, Sonoyama K. Exosomes isolated from sera of mice fed *lactobacillus* strains affect inflammatory cytokine production in macrophages in vitro. Biochem Biophys Res Commun. 2017;489:248–254. doi:10.1016/j.bbrc.2017.05.152. 28559134

[47] Forsythe P, Bienenstock J, Kunze WA. Vagal pathways for microbiome-brain-gut axis communication. Adv Exp Med Biol. 2014;817:115–133. 24997031 10.1007/978-1-4939-0897-4_5

[48] Perez-Burgos A, Mao Y-K, Bienenstock J, Kunze WA. The gut-brain axis rewired: adding a functional vagal nicotinic “sensory synapse”. FASEB J. 2014;28:3064–3074. 24719355 10.1096/fj.13-245282

[49] Wang B, Mao Y-K, Diorio C, Pasyk M, Ry W, Bienenstock J, Wa K. Luminal administration ex vivo of a live *lactobacillus* species moderates mouse jejunal motility within minutes. FASEB J. 2010;24:4078–4088. doi:10.1096/fj.09-153841. 20519636

[50] Parish CR. Fluorescent dyes for lymphocyte migration and proliferation studies. Immunol Cell Biol. 1999;77(6):499–508. doi:10.1046/j.1440-1711.1999.00877.x. 10571670

[51] Biller SJ, Schubotz F, Roggensack SE, Thompson AW, Summons RE, Chisholm SW. Bacterial vesicles in marine ecosystems. Science. 2014;343(6167):183–186. doi:10.1126/science.1243457. 24408433

[52] Toyofuku M, Nomura N, Eberl L. Types and origins of bacterial membrane vesicles. Nat Rev Microbiol. 2019;17(1):13–24. doi:10.1038/s41579-018-0112-2. 30397270

[53] Toyofuku M, Cárcamo-Oyarce G, Yamamoto T, Eisenstein F, Hsiao -C-C, Kurosawa M, Gademann K, Pilhofer M, Nomura N, Eberl L Prophage-triggered membrane vesicle formation through peptidoglycan damage in bacillus subtilis. Nat Commun [Internet]; 2017 [cited 2020 Jan 24]; 8. Available from: https://www.ncbi.nlm.nih.gov/pmc/articles/PMC5589764/10.1038/s41467-017-00492-w PMC5589764 28883390

[54] Bichet MC, Chin WH, Richards W, Lin Y-W, Avellaneda-Franco L, Hernandez CA, Oddo A, Chernyavskiy O, Hilsenstein V, Neild A. Bacteriophage uptake by mammalian cell layers represents a potential sink that may impact phage therapy. iScience. 2021;24(4):102287. doi:10.1016/j.isci.2021.102287. 33855278 PMC8024918

[55] Nguyen S, Baker K, Padman BS, Patwa R, Dunstan RA, Weston TA, Schlosser K, Bailey B, Lithgow T, Lazarou M. Bacteriophage transcytosis provides a mechanism to cross epithelial cell layers. mBio. 2017;8(6):e01874–17. doi:10.1128/mBio.01874-17. 29162715 PMC5698557

[56] Blanco-Picazo P, Fernández-Orth D, Brown-Jaque M, Miró E, Espinal P, Rodríguez-Rubio L, Muniesa M, Navarro F Unravelling the consequences of the bacteriophages in human samples. Sci Rep [Internet]; 2020 [cited 2021 Mar 11]; 10. Available from: https://www.ncbi.nlm.nih.gov/pmc/articles/PMC7174282/10.1038/s41598-020-63432-7 PMC7174282 32317653

[57] Keller R, Engley FB. Fate of bacteriophage particles introduced into mice by various routes. Proc Soc Exp Biol Med. 1958;98:577–580. doi:10.3181/00379727-98-24112. 13567777

[58] Popescu M, Van Belleghem JD, Khosravi A, Bollyky PL Bacteriophages and the immune system. Annu Rev Virol [Internet]; 2021 [cited 2021 May 26]. Available from: http://www.annualreviews.org/doi/10.1146/annurev-virology-091919-074551 34014761

[59] Gogokhia L, Buhrke K, Bell R, Hoffman B, Brown DG, Hanke-Gogokhia C, Ajami NJ, Wong MC, Ghazaryan A, Valentine JF. Expansion of bacteriophages is linked to aggravated intestinal inflammation and colitis. Cell Host Microbe. 2019;25(2):e8. doi:10.1016/j.chom.2019.01.008. PMC6885004 30763538

[60] Sweere JM, Belleghem JDV, Ishak H, Bach MS, Popescu M, Sunkari V, Kaber G, Manasherob R, Suh GA, Cao X. Bacteriophage trigger antiviral immunity and prevent clearance of bacterial infection. Science. 2019;363(6434):eaat9691. doi:10.1126/science.aat9691. 30923196 PMC6656896

[61] Shepardson KM, Schwarz B, Larson K, Morton RV, Avera J, McCoy K, Caffrey A, Harmsen A, Douglas T, Rynda-Apple A Induction of antiviral immune response through recognition of the repeating subunit pattern of viral capsids is toll-like receptor 2 dependent. mBio [Internet]; 2017 [cited 2021 May 28]; 8. Available from: https://www.ncbi.nlm.nih.gov/pmc/articles/PMC5686532/10.1128/mBio.01356-17 PMC5686532 29138299

