# Supplementary material for: Bacterial membrane vesicles and phages in blood after consumption of lacticaseibacillus rhamnosus JB-1
Source: Gut Microbes. 2021 Nov 7;13(1):1993583. doi: 10.1080/19490976.2021.1993583 (PMC8583084; doi:10.1080/19490976.2021.1993583)
Supplement: Supplemental Material [file KGMI_A_1993583_SM4801.pdf]

Supplementary Information for

Bacterial membrane vesicles and phages in blood after consumption of *Lacticaseibacillus rhamnosus* JB-1

Kevin Champagne-Jorgensen[1,2,#], Tamina A. Jose[3,4], Andrew M. Stanisz[2], M. Firoz Mian[2], Alexander P. Hynes[4,5,*], John Bienenstock[2,6,*]

[1] Neuroscience Graduate Program, McMaster University, Hamilton, ON L8S 4L8, Canada
[2] Brain-Body Institute, St. Joseph's Healthcare Hamilton, Hamilton, ON L8N 4A6, Canada
[3] Department of Biochemistry and Biomedical Sciences, McMaster University, Hamilton, ON L8S 4L8, Canada
[4] Farncombe Family Digestive Health Research Institute, Department of Medicine, McMaster University, Hamilton, ON L8S 4L8, Canada
[5] Department of Medicine, McMaster University, Hamilton, ON L8S 4L8, Canada
[6] Department of Pathology and Molecular Medicine, McMaster University, Hamilton, ON L8S 4L8, Canada
[*] These authors contributed equally
[#] Corresponding author: champk1@mcmaster.ca

Supplementary figures

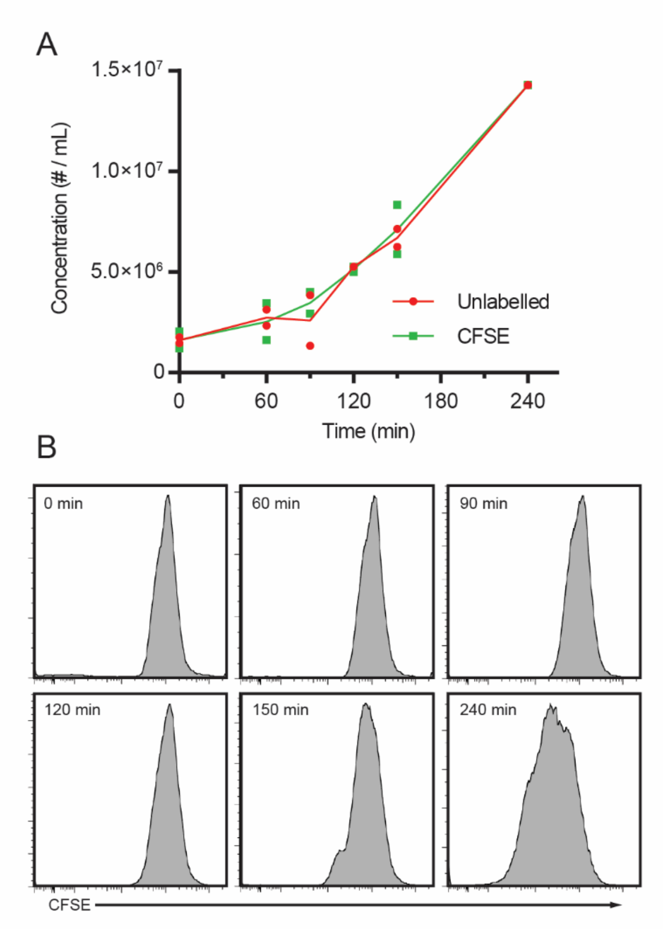

Figure S1. Fluorescence labelling with CFSE does not alter growth of *L. rhamnosus* JB-1. Bacteria were labelled with 20 µM CFSE, washed, then cultured in duplicate in 45 mL MRS broth. Duplicate unstained control JB-1 cultures were initiated identically. Cultures were sampled after 0, 60, 90, 120, 150, and 240 minutes in culture, fixed with 4% formaldehyde, then enumerated by flow cytometry with a flow rate of 60 µL/min. (**A**) Concentrations of bacteria at each time point were estimated by the amount of time it took to count 100,000 particles and are plotted above. (**B**) CFSE fluorescence across time for one representative replicate.

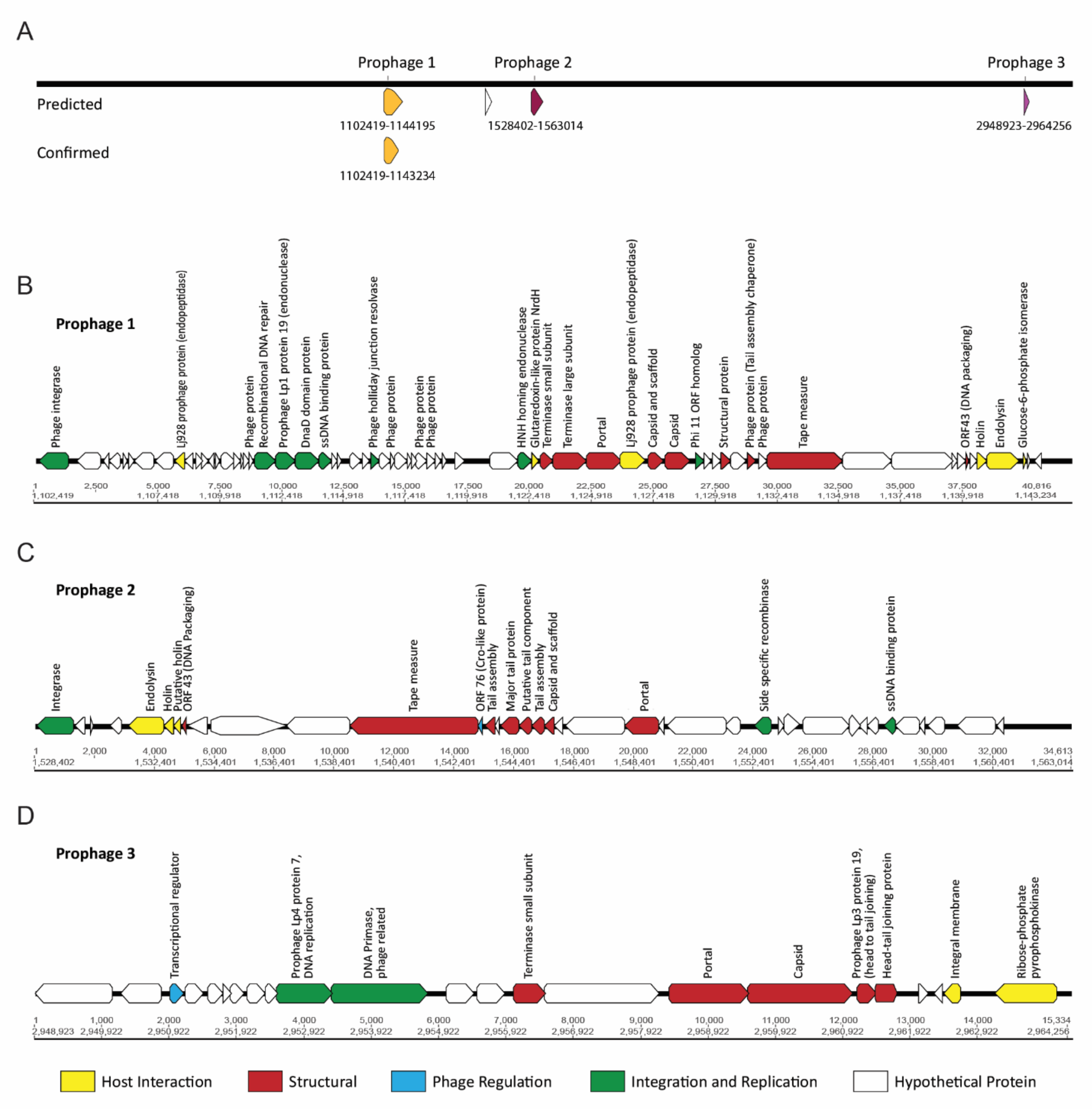

Figure S2. Bioinformatic tool predicts prophages in *L. rhamnosus* GG. (**A**) Phaster was used to identify prophages within the *L. rhamnosus* GG genome as a proxy for *L. rhamnosus* JB-1. Predictions were manually curated based on the presence of signature phage genes, such as capsid, terminase, and tail structural modules. A total of 4 prophages were predicted, of which 3 carry phage specific genes. (**B**-**D**) Designated prophages were then annotated in detail. Identified open reading frames and directions are represented by coloured arrows and predicted gene names are labelled as indicated for (**B**) Prophage 1, (**C**) Prophage 2, and (**D**) Prophage 3.

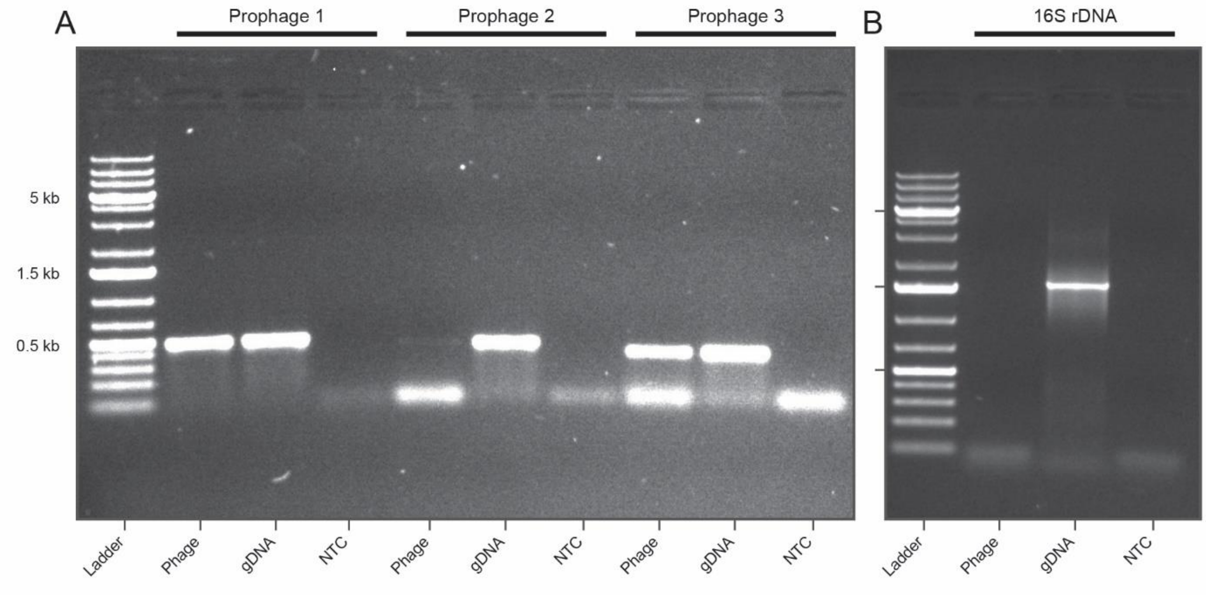

Figure S3. Three prophages are released after induced DNA damage. Mitomycin C was used to damage bacterial DNA and induce phage release. Phages were then precipitated from the culture supernatant by polyethylene glycol. Precipitates were sequentially treated with nucleases and proteinase K prior to DNA extraction. Precipitated DNA extracts (Phage), *L. rhamnosus* JB-1 genomic DNA extract (gDNA), or no template controls (NTC) were amplified by PCR for (**A**) indicated prophage sequences or (**B**) universal 16S rDNA sequence, then were visualized after electrophoresis.

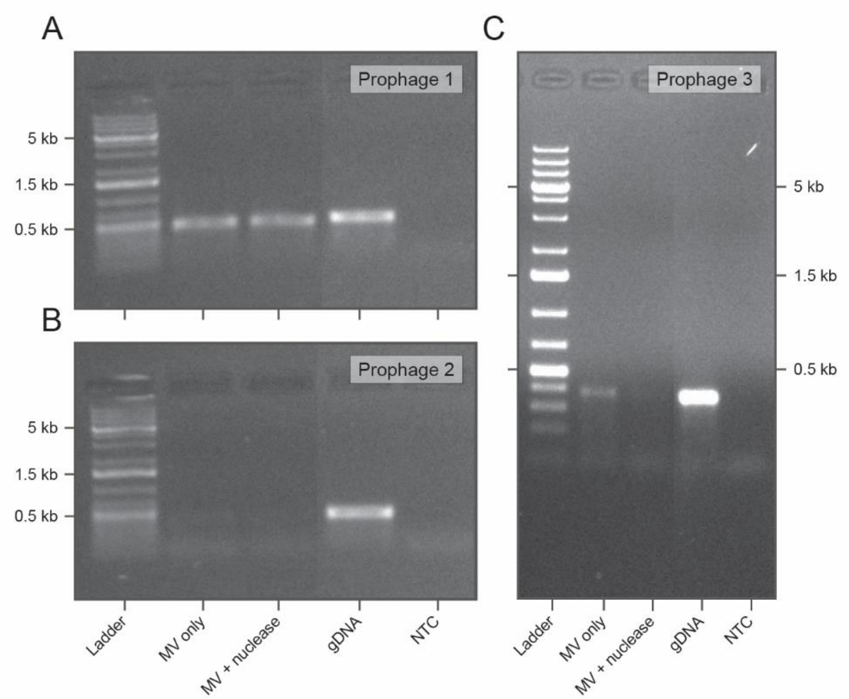

Figure S4. Prophage 1 is released by JB-1 in the absence of applied stressors. JB-1 MV were collected from ultracentrifuged culture supernatants (MV only) and treated with DNase and RNase (MV + nuclease). Extracted DNA from these, JB-1 bacteria (gDNA), or no template control (NTC) were then tested by PCR for (**A**) Prophage 1 DNA, (**B**) Prophage 2 DNA, and (**C**) Prophage 3 DNA, and visualized after DNA electrophoresis. Note that gels are cropped to remove unrelated wells between nuclease and gDNA wells from the same gel.

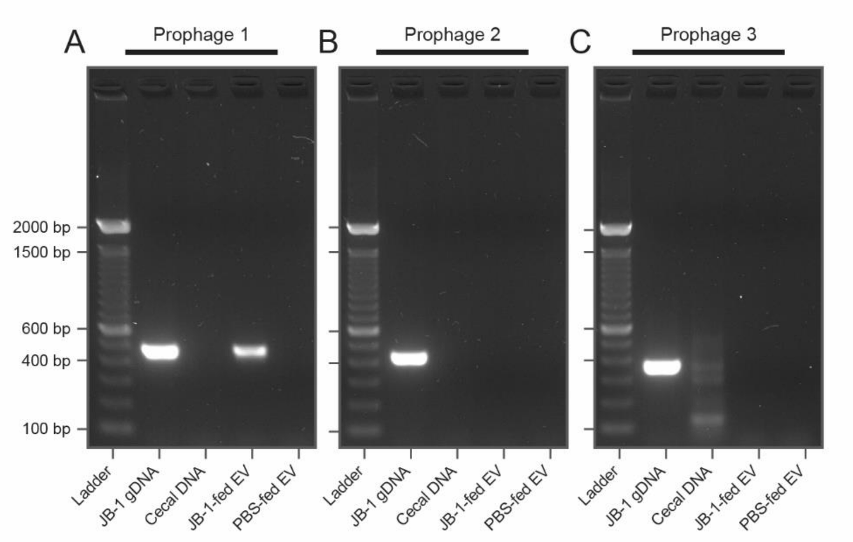

Figure S5. EV isolated from plasma of mice fed *L. rhamnosus* JB-1 contain phage DNA (full-length gels). DNA was isolated from JB-1 bacteria, naïve mouse cecal contents, EV from JB-1-fed mice, or EV from PBS-fed mice, then subject to qPCR and products electrophoresed. Gels depict PCR products amplified by (**A**) Prophage 1 primers, (**B**) Prophage 2 primers, and (**C**) Prophage 3 primers.

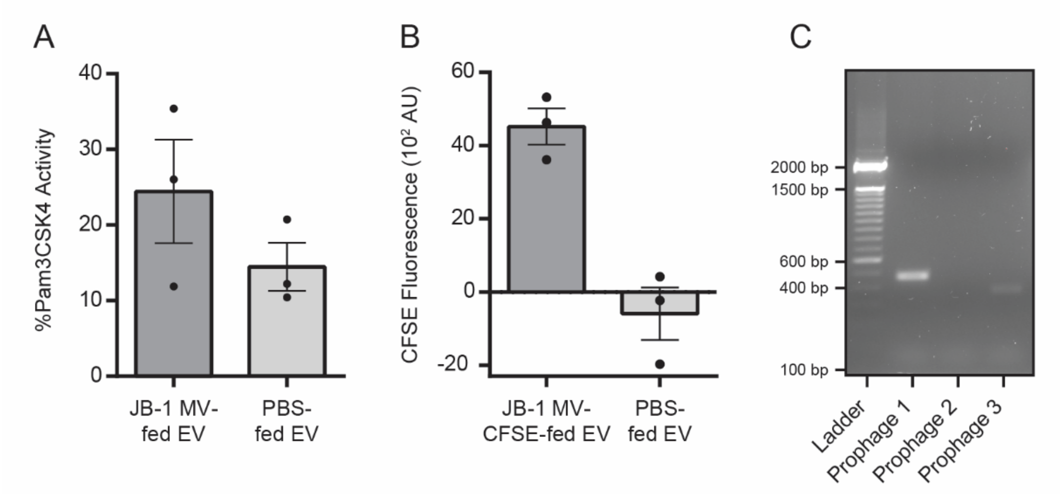

Figure S6. EV isolated from plasma of mice fed MV of *L. rhamnosus* JB-1 contain bacterial components. Mice were gavaged approximately 3 × 10^{10} JB-1 MV in 200 µL PBS or PBS alone and plasma EV were then assessed for bacterial components. (**A**) TLR2 activation by EV was quantified by reporter cell line, with data expressed as a percentage of activity measured for the synthetic TLR2 ligand Pam3CSK4 (300 ng/mL). Each point represents one EV preparation (3-6 mice pooled). (**B**) Plasma EV from mice fed with CFSE-labelled JB-1 MV or PBS vehicle were assessed for CFSE-related fluorescence using a plate reader. Data are shown after subtraction of PBS blank wells. (**C**) DNA electrophoresis of qPCR products from isolated DNA showing Prophage 1 (average 33 cycles) and Prophage 3 (average 35 cycles) DNA, but not Prophage 2 DNA, were present in EV from JB-1 MV-fed mice.

Supplementary tables

Table S1. Proteins differentially expressed in plasma EV from mice fed *L. rhamnosus* JB-1 relative to EV from mice fed PBS.

Fold Change*	p-value	Accession	Gene	Description
51.38	0.0115	Q5XK03	Serinc4	Serine incorporator 4
16.34	0.0486	P06795	Abcb1b	Multidrug resistance protein 1B
13.55	0.0197	E9PZQ0	Ryr1	Ryanodine receptor 1
11.95	0.0089	Q9JJR8	Tmem9b	Transmembrane protein 9B
8.75	0.0298	Q80Y19	Arhgap11a	Rho GTPase-activating protein 11A
7.98	0.0156	Q80X50	Ubap2l	Ubiquitin-associated protein 2-like
6.89	0.0114	Q7TSQ1	Clec18a	C-type lectin domain family 18 member A
6.63	0.0221	P19070	Cr2	Complement receptor type 2
5.98	0.0204	P97499	Tep1	Telomerase protein component 1
5.53	0.0476	Q9EQJ0	Tpcn1	Two pore calcium channel protein 1
4.98	0.0381	Q8C0R0	Usp37	Ubiquitin carboxyl-terminal hydrolase 37
4.89	0.0388	Q9ESG9	Pkmyt1	Myt1 kinase
4.21	0.0438	P35922	Fmr1	Synaptic functional regulator FMR1
4.15	0.0497	P01865	Igh-1a	Ig gamma-2A chain C region membrane-bound form
3.94	0.0473	Q80WJ6	Abcc12	Multidrug resistance-associated protein 9
3.54	0.0301	Q6ZQF0	Topbp1	DNA topoisomerase 2-binding protein 1
3.26	0.0086	P55066	Ncan	Neurocan core protein
3.19	0.0221	Q9EPQ2	Rpgrip1	RPGR-interacting protein 1
2.89	0.0356	P01869	Ighg1	Ig gamma-1 chain C region membrane-bound form
-3.85	0.0071	P33622	Apoc3	Apolipoprotein C-III
-4.44	0.0443	P11859	Agt	Angiotensinogen
-5.44	0.0075	Q6GQT1	A2m	Alpha-2-macroglobulin-P
-6.26	0.0484	Q8BHB0	Nod1	Nucleotide-binding oligomerization domain-containing protein 1
-6.40	0.0276	Q80YT7	Pde4dip	Myomegalin
-7.29	0.0258	O70279	Ess2	Splicing factor ESS-2 homolog

*Fold change expressed as average protein intensity in JB-1-fed EV divided by average protein intensity in PBS-fed EV; if less than 1, then result is multiplied by the negative reciprocal

Table S2. Proteins found exclusively in plasma EV from mice fed either *L. rhamnosus* JB-1 or PBS.

Found in	Accession	Gene	Description
JB-1 EV	Q8VEC3	Adgrf1	Adhesion G-protein coupled receptor F1
JB-1 EV	Q766D5	B4galnt4	Beta-1,4-N-acetylgalactosaminyltransferase IV
JB-1 EV	Q8K2J9	Btbd6	BTB/POZ domain-containing protein 6
JB-1 EV	P10287	Cdh3	Cadherin-3
JB-1 EV	O70566	Diaph2	Protein diaphanous homolog 2
JB-1 EV	Q69ZL1	Fgd6	FYVE RhoGEF and PH domain-containing protein 6
JB-1 EV	P52187	Kcnj12	ATP-sensitive inward rectifier potassium channel 12
JB-1 EV	Q6P5G3	Mbtd1	MBT domain-containing protein 1
JB-1 EV	Q0II04	Nebl	Nebulette
JB-1 EV	Q923S6	Neurl1	E3 ubiquitin-protein ligase NEURL1
JB-1 EV	O54803	P2rx6	P2X purinoceptor 6
JB-1 EV	Q1EHW4	Sap25	Histone deacetylase complex subunit SAP25
JB-1 EV	Q6ZPE2	Sbf1	Myotubularin-related protein 5
JB-1 EV	Q61420	Slc35a1	CMP-sialic acid transporter
JB-1 EV	Q8R1X6	Spart	Spartin
JB-1 EV	Q8K2X3	Stn1	CST complex subunit STN1
JB-1 EV	Q9ES63	Usp29	Ubiquitin carboxyl-terminal hydrolase 29
JB-1 EV	P24383	Wnt7a	Protein Wnt-7a
JB-1 EV	Q3V0E1	N/A	Uncharacterized protein C9orf131 homolog
PBS EV	Q9JIY2	Cbll1	E3 ubiquitin-protein ligase Hakai
PBS EV	Q8C7V8	Ccdc134	Coiled-coil domain-containing protein 134
PBS EV	Q8BWD8	Cdk19	Cyclin-dependent kinase 19
PBS EV	Q8BWY9	Cip2a	Protein CIP2A
PBS EV	Q2UY11	Col28a1	Collagen alpha-1(XXVIII) chain
PBS EV	Q9WVJ3	Cpq	Carboxypeptidase Q
PBS EV	Q6P5D3	Dhx57	Putative ATP-dependent RNA helicase DHX57
PBS EV	Q99MS7	Ehbp1l1	EH domain-binding protein 1-like protein 1
PBS EV	P63017	Hspa8	Heat shock cognate 71 kDa protein
PBS EV	Q9WV04	Kif9	Kinesin-like protein KIF9
PBS EV	Q9JHQ5	Lztfl1	Leucine zipper transcription factor-like protein 1
PBS EV	P13297	Msx1	Homeobox protein MSX-1
PBS EV	Q9CQH3	Ndufb5	NADH-ubiquinone oxidoreductase SGDH subunit
PBS EV	Q60890	Olfr11	Olfactory receptor 11
PBS EV	Q63836	Selenbp2	Selenium-binding protein 2
PBS EV	Q9D7D2	Serpina9	Serpin A9
PBS EV	Q9QUK6	Tlr4	Toll-like receptor 4

Table S3. GO terms enriched in proteins upregulated in EV from mice fed *L. rhamnosus* JB-1.

*	GO ID	GO Term	FDR	# Genes	Total Genes	Genes
BP	0099565	Chemical synaptic transmission, postsynaptic	0.022	4	114	GLRA4, P2RX6, WNT7A, GRIN1
BP	0007010	Cytoskeleton organization	0.022	13	1288	NEK2, RHOH, NEBL, TRF, DIAPH2, SORBS1, ARHGAP26, CEP250, SIPA1L1, CENPE, NPHS1, SPAG5, NEURL1A
BP	0014045	Establishment of endothelial blood-brain barrier	0.022	2	5	WNT7A, ABCB1B
BP	0022607	Cellular component assembly	0.022	20	2792	CLU, NEK2, NEBL, CDH3, NEURL1A, WNT7A, DIAPH2, FMR1, P2RX6, LAMC1, ICE1, CEP250, CENPE, NPHS1, GRIN1, ADGRF1, SPAG5, OPTN, KCNJ12, SIPA1L1
BP	0043087	Regulation of GTPase activity	0.022	7	433	PLXNA1, FGD6, ARHGAP26, SBF1, ARHGAP11A, SIPA1L1, ARHGAP8
BP	0044093	Positive regulation of molecular function	0.022	15	1654	TRF, ADGRF1, FMR1, PSMA6, CLU, NEK2, ARHGAP26, ARHGAP11A, SIPA1L1, CENPE, CDH3, ARHGAP8, MAGI3, NEURL1A, GRIN1
BP	0050770	Regulation of axonogenesis	0.022	5	193	PLXNA1, WNT7A, SIPA1L1, SPG20, GRIN1
BP	0051128	Regulation of cellular component organization	0.022	19	2487	WNT7A, CLU, NEK2, RHOH, PLXNA1, STN1, NEURL1A, SIPA1L1, FMR1, OPTN, ICE1, CEP250, CENPE, NPHS1, SPG20, GRIN1, SPAG5, TRF, SBF1
BP	0051983	Regulation of chromosome segregation	0.022	4	103	SPAG5, NEK2, PUM1, CENPE
BP	0060538	Skeletal muscle organ development	0.022	5	185	NEURL1A, NPHS1, RBFOX1, MYT1, PSMA6
BP	0065009	Regulation of molecular function	0.022	21	3108	PLXNA1, SERPINA3M, NEK2, TRF, ADGRF1, FMR1, FGD6, PSMA6, CLU, RHOH, WNT7A, ARHGAP26, SBF1, ARHGAP11A, SIPA1L1, CENPE, CDH3, ARHGAP8, MAGI3, NEURL1A, GRIN1
BP	0051130	Positive regulation of cellular component organization	0.024	12	1258	PLXNA1, NEURL1A, WNT7A, FMR1, CLU, NEK2, OPTN, ICE1, NPHS1, SPAG5, GRIN1, TRF
BP	0044085	Cellular component biogenesis	0.030	20	3000	CLU, NEK2, NEBL, CDH3, NEURL1A, WNT7A, DIAPH2, FMR1, P2RX6, LAMC1, ICE1, CEP250, CENPE, NPHS1, GRIN1, ADGRF1, SPAG5, OPTN, KCNJ12, SIPA1L1
BP	0030036	Actin cytoskeleton organization	0.030	8	640	RHOH, NEBL, TRF, DIAPH2, SORBS1, ARHGAP26, SIPA1L1, NPHS1
BP	1904889	Regulation of excitatory synapse assembly	0.038	2	17	WNT7A, SIPA1L1
BP	0060078	Regulation of postsynaptic membrane potential	0.038	4	149	GLRA4, P2RX6, WNT7A, GRIN1
BP	0048468	Cell development	0.046	16	2307	PLXNA1, NEBL, NEURL1A, WNT7A, ADGRF1, SIPA1L1, FMR1, NPHS1, CLU, LAMC1, DIAPH2, RPGRIP1, SPG20, GRIN1, ABCB1B, TRF
BP	0061351	Neural precursor cell proliferation	0.046	4	168	WNT7A, RPGRIP1, OPTN, TRF
BP	2000637	Positive regulation of gene silencing by miRNA	0.047	2	24	FMR1, PUM1
BP	0032989	Cellular component morphogenesis	0.047	10	1118	RHOH, PLXNA1, NEBL, CDH3, WNT7A, SIPA1L1, CLU, LAMC1, SPG20, GRIN1
BP	0044087	Regulation of cellular component biogenesis	0.047	9	931	CLU, NEURL1A, WNT7A, FMR1, ICE1, NPHS1, GRIN1, SPAG5, SIPA1L1
CC	0030054	Cell junction	0.006	13	1095	MAGI1, MAGI3, CDH3, NPHS1, ABCB1B, NEURL1A, GLRA4, P2RX6, SORBS1, GRIN1, ARHGAP26, SIPA1L1, PRR12
CC	0005604	Basement membrane	0.034	3	99	FREM2, LAMC1, TRF
CC	0005819	Spindle	0.034	5	341	SPAG5, NEK2, CEP250, TOPBP1, CENPE
CC	0005856	Cytoskeleton	0.034	15	2123	NEK2, SPAG5, CEP250, RPGRIP1, FGD6, CLU, SORBS1, TOPBP1, ARHGAP26, KRT80, STN1, SIPA1L1, CENPE, KRT42, NEBL
CC	0005912	Adherens junction	0.034	5	304	CDH3, NPHS1, MAGI1, SORBS1, ARHGAP26
CC	0030496	Midbody	0.034	4	176	NEK2, SPAG5, SPG20, CENPE
CC	0042995	Cell projection	0.034	16	2328	GLRA4, RHOH, NEURL1A, NPHS1, GRIN1, RPGRIP1, FMR1, CLU, P2RX6, CEP250, SIPA1L1, MAGI1, PRR12, ALS2CR12, OPTN, KCNJ12
CC	0043025	Neuronal cell body	0.034	8	684	NEURL1A, FMR1, GLRA4, P2RX6, OPTN, GRIN1, KCNJ12, SIPA1L1
CC	0043197	Dendritic spine	0.034	4	204	NEURL1A, GRIN1, P2RX6, SIPA1L1
CC	0048471	Perinuclear region of cytoplasm	0.034	8	748	OPTN, FMR1, NEURL1A, CLU, DOCK6, SBF1, CEP250, TRF
CC	0097060	Synaptic membrane	0.034	6	485	GLRA4, GRIN1, NEURL1A, P2RX6, SIPA1L1, PRR12
CC	0097440	Apical dendrite	0.034	2	25	NEURL1A, CLU
CC	0098688	Parallel fiber to Purkinje cell synapse	0.034	2	27	P2RX6, GRIN1

CC	0099572	Postsynaptic specialization	0.034	6	423	NEURL1A, GLRA4, GRIN1, P2RX6, SIPA1L1, PRR12
CC	0000779	Condensed chromosome, centromeric region	0.036	3	110	SPAG5, NEK2, CENPE
CC	0043235	Receptor complex	0.044	5	377	GRIN1, PLXNA1, SORBS1, CR2, TRF
CC	0032279	Asymmetric synapse	0.045	5	390	NEURL1A, GRIN1, P2RX6, SIPA1L1, PRR12

*GO Aspect: PB = Biological Process; CC = Cellular Component

Table S4. *L. rhamnosus* GG Prophage 1 annotation and best phage hit values.

Name	AA Length	Predicted Product	Best Phage Hit (E-value)	% Identity, % Coverage
P1Lr1	395	Phage integrase	Lactobacillus phage JNU_P10 (0.0)	97.89%, 100%
P1Lr2	334	Hypothetical protein		
P1Lr3	68	Hypothetical protein	Lactobacillus phage JNU_P10 (3e-67)	90.20%, 100%
P1Lr4	142	Hypothetical protein	Lactobacillus phage PLE3 (0.0)	97.42%, 100%
P1Lr5	56	Hypothetical protein	Lactobacillus phage JNU_P10 (1e-80)	100%, 100%
P1Lr6	74	Hypothetical protein	Lactobacillus phage JNU_P10 (6e-109)	99.55%, 100%
P1Lr7	252	Hypothetical protein	Lactobacillus phage JNU_P10 (0.0)	100%, 100%
P1Lr8	234	Hypothetical protein	Lactobacillus phage JNU_P10 (0.0)	91.31%, 100%
P1Lr9	141	Lj928 prophage protein	Lactobacillus phage iLp84 (5e-143)	88.89%, 99%
P1Lr10	112	Hypothetical protein	Lactobacillus phage iLp84 (1e-143)	94.64%, 100%
P1Lr11	81	Hypothetical protein	Lactobacillus phage iLp84 (7e-104)	95.47%, 100%
P1Lr12	83	Hypothetical protein		
P1Lr13	76	Hypothetical protein		
P1Lr14	53	Hypothetical protein		
P1Lr15	183	Hypothetical protein	Lactobacillus phage PLE3 (0.0)	97.09%, 100%
P1Lr16	77	Hypothetical protein	Lactobacillus phage PLE3 (3e-78)	92.09%, 93%
P1Lr17	43	Hypothetical protein		
P1Lr18	76	Hypothetical protein		
P1Lr19	57	Phage protein	Lactobacillus phage Lc-Nu (5e-79)	98.84%, 100%
P1Lr20	292	Recombinational DNA repair protein RecT (prophage associated)	Lactobacillus phage Lc-Nu (2e-20)	100%, 6%
P1Lr21	255	Prophage Lp1 protein 19		
P1Lr22	319	DnaD domain protein	Lactobacillus phage PLE3 (0.0)	95.69%, 94%
P1Lr23	162	Single-stranded DNA-binding protein		
P1Lr24	72	Hypothetical protein		
P1Lr25	64	Hypothetical protein		
P1Lr26	150	Hypothetical protein		
P1Lr27	85	Hypothetical protein		
P1Lr28	114	Phage Holliday junction resolvase	Lactobacillus phage Lc-Nu (2e-50)	80.77%, 81%
P1Lr29	155	Hypothetical protein		
P1Lr30	62	Phage protein	Lactobacillus phage T25 (4e-70)	94.41%, 96%
P1Lr31	169	Hypothetical protein	Lactobacillus casei bacteriophage A2 (5e-104)	86.5%, 71%
P1Lr32	60	Hypothetical protein	Lactobacillus phage BH1 (7e-63)	93.14%, 95%
P1Lr33	68	Hypothetical protein		
P1Lr34	144	Phage protein	Lactobacillus virus Lb338-1 (1e-83)	86.64%, 74%
P1Lr35	121	Phage protein	Lactobacillus phage Lrm1 (1e-19)	88.17%, 25%
P1Lr36	80	Hypothetical protein		
P1Lr37	61	Hypothetical protein	Lactobacillus phage phiAT3 (3e-21)	83.20%, 68%
P1Lr38	143	Hypothetical protein		
P1Lr39	383	Hypothetical protein	Lactobacillus phage Lrm1 (0.0)	97.57%, 100%
P1Lr40	181	HNH homing endonuclease	Lactobacillus phage Lrm1 (0.0)	100%, 100%
P1Lr41	107	Glutaredoxin-like protein NrdH	Lactobacillus phage Lc-Nu (8e-105)	88.99%, 99%

P1Lr42	178	Phage terminase, small subunit	Lactobacillus phage PLE3 (0.0)	94.98%, 85%
P1Lr43	452	Terminase large subunit (Bacteriophage A118)		
P1Lr44	476	Phage portal protein	Lactobacillus phage PLE3 (0.0)	88.36%, 100%
P1Lr45	331	Lj928 prophage protein	Lactobacillus phage PLE3 (0.0)	93.66%, 100%
P1Lr46	219	Phage capsid and scaffold	Lactobacillus phage JNU_P10 (0.0)	92.62%, 82%
P1Lr47	338	Phage capsid protein		
P1Lr48	125	phi 11 orf36 homolog [SA bacteriophages 11, Mu50B]	Lactobacillus phage PLE3 (2e-150)	92.8%, 100%
P1Lr49	101	Hypothetical protein	Lactobacillus phage PLE3 (2e-120)	93.07%, 100%
P1Lr50	114	Hypothetical protein	Lactobacillus phage PLE3 (1e-133)	93.83%, 100%
P1Lr51	135	Structural protein	Lactobacillus phage PLE3 (0.0)	95.80%, 100%
P1Lr52	201	Hypothetical protein	Lactobacillus phage iLp1308 (0.0)	89.60%, 100%
P1Lr53	111	Phage protein	Lactobacillus phage iLp1308 (5e-137)	93.69%, 100%
P1Lr54	118	Phage protein	Lactobacillus phage iLp1308 (2e-140)	92.66%, 100%
P1Lr55	1030	Phage tape measure	Lactobacillus phage JNU_P10 (0.0)	91.83%, 98%
P1Lr56	663	Hypothetical protein	Lactobacillus phage Lrm1 (0.0)	98.66%, 78%
P1Lr57	836	Hypothetical protein	Lactobacillus phage Lrm1 (0.0)	97.88%, 100%
P1Lr58	108	Hypothetical protein	Lactobacillus phage Lrm1 (4e-162)	99.07%, 100%
P1Lr59	44	Orf43	Lactobacillus phage Lrm1 (8e-61)	100.00%, 100%
P1Lr60	98	Hypothetical protein YeeN	Lactobacillus phage Lrm1 (7e-125)	96.73%, 93%
P1Lr61	138	Holin	Lactobacillus phage BH1 (0.0)	94.93%, 100%
P1Lr62	433	Phage endolysin	Lactobacillus phage Lrm1 (0.0)	98.15%, 100%
P1Lr63	38	Glucose-6-phosphate isomerase (EC 5.3.1.9)	Lactobacillus phage JNU_P10 (1e-42)	95.61%, 100%
P1Lr64	44	Hypothetical protein		
P1Lr65	96	Hypothetical protein		
P1Lr66	248	DNA-entry nuclease (Competence-specific nuclease) (EC 3.1.30)		

Table S5. *L. rhamnosus* GG Prophage 2 annotation and best phage hit values.

Name	AA Length	Predicted Product	Best Phage Hit (E-value)	% Identity, % Coverage
P2Lr1	390	Integrase, superantigen-encoding pathogenicity islands SaPI		
P2Lr2	109	Hypothetical protein		
P2Lr3	40	Hypothetical protein		
P2Lr4	129	Hypothetical protein		
P2Lr5	385	Endolysin	Lactobacillus phage T25 (9e-80)	73.31%, 78%
P2Lr6	111	Phage holin		
P2Lr7	72	Putative holin		
P2Lr8	44	Orf43	Lactobacillus phage T25 (8e-46)	93.18%, 100%
P2Lr9	108	Hypothetical protein	Lactobacillus phage Lrm1 (2e-160)	98.77%, 100%
P2Lr10	978	Hypothetical protein	Lactobacillus phage Lrm1 (0.0)	91.80%, 59%
P2Lr11	686	Hypothetical protein	Lactobacillus phage J-1 (0.0)	87.50%, 31%
P2Lr12	1,392	Phage tail length tape-measure protein T CDS	Lactobacillus phage Lc-Nu (0.0)	92.91%, 76%
P2Lr13	52	Orf76 CDS	Lactobacillus rhamnosus Lc-Nu-like prophage (4e-69)	98.08%, 100%
P2Lr14	117	Phage tail assembly CDS	Lactobacillus phage T25 (2e-145)	93.73%, 100%
P2Lr15	212	Phage major tail protein	Lactobacillus phage T25 (0.0)	94.79%, 96%
P2Lr16	127	Putative tail component	Lactobacillus rhamnosus Lc-Nu-like prophage (1e-148)	92.13%, 100%
P2Lr17	140	Phage tail assembly	Lactobacillus rhamnosus Lc-Nu-like prophage (1e-139)	88.63%, 99%
P2Lr18	115	Phage capsid and scaffold	Lactobacillus phage Lc-Nu (9e-75)	83.99%, 88%
P2Lr19	97	Hypothetical protein		
P2Lr20	643	Hypothetical protein		
P2Lr21	367	Phage portal protein		
P2Lr22	61	Hypothetical protein		
P2Lr23	631	Hypothetical protein		
P2Lr24	155	Hypothetical protein		
P2Lr25	187	Site-specific recombinase, DNA invertase Pin related protein		
P2Lr26	59	Hypothetical protein		
P2Lr27	250	Hypothetical protein		
P2Lr28	472	Hypothetical protein		
P2Lr29	101	Hypothetical protein		
P2Lr30	93	Hypothetical protein		
P2Lr31	181	Hypothetical protein		
P2Lr32	135	Single-stranded DNA-binding protein		
P2Lr33	256	Hypothetical protein		
P2Lr34	69	Hypothetical protein		
P2Lr35	194	Hypothetical protein		
P2Lr36	413	Hypothetical protein		
P2Lr37	76	Hypothetical protein		

Table S6. ***L. rhamnosus*** **GG Prophage 3 annotation**.

Name	AA Length	Predicted Product
P3Lr1	386	Hypothetical protein
P3Lr2	204	Hypothetical protein
P3Lr3	71	Transcriptional regulator, putative CDS
P3Lr4	92	Hypothetical protein
P3Lr5	74	Hypothetical protein YeeN
P3Lr6	45	Hypothetical protein
P3Lr7	64	Hypothetical protein YeeN
P3Lr8	91	Hypothetical protein
P3Lr9	63	Hypothetical protein
P3Lr10	276	Prophage Lp4 protein 7, DNA replication CDS
P3Lr11	475	DNA primase, phage associated CDS
P3Lr12	141	Hypothetical protein
P3Lr13	140	Hypothetical protein
P3Lr14	157	Phage terminase small subunit CDS
P3Lr15	568	Hypothetical protein
P3Lr16	395	Phage portal protein CDS
P3Lr17	520	Phage capsid protein CDS
P3Lr18	97	Prophage Lp3 protein 19, head-to-tail joining CDS
P3Lr19	72	Putative head-tail joining protein CDS
P3Lr20	46	Hypothetical protein
P3Lr21	47	Hypothetical protein
P3Lr22	83	Integrse CDS
P3Lr23	307	Ribose-phosphate pyrophosphokinase (EC 2.7.6.1)

Table S7. Primers used in this study.

Entity	Gene	Forward Primer Sequence	Reverse Primer Sequence
Prophage 1	Terminase large subunit	TAGACTGGATGAGGTGCCAA	ACGTGTGGATCGTCAGAATG
Prophage 2	Phage portal protein	GGAACACCAAAAACCTTGCC	GCAGACAGCAGTGCAGATAT
Prophage 3	Phage capsid protein	AAGAAACAGGACGACACCAC	TACGAATGACTTGTCCGCTG
Bacterial 16S	16S rRNA	AGAGTTTGATCCTGGCTCAG	GGTTACCTTGTTACGACTT
